# Analectic

**Published:** 1831

**Authors:** 


### MISCELLANEOUS INTELLIGENCE.

### ANALECTIC, ANALYTICAL, AND ORIGINAL.

### I. ANALECTIC.

1. *Singular Trial for Infanticide.*—Mr. Beasse, a person of tolerable station on the Island of Guernsey, carried on an illicit intercourse with his servant, Sarah Elliott, the consequence of which was that she became pregnant. Early in the month of May he mentioned the latter circumstance to a friend, and confided to him his intention of having the girl sent across to the adjoining coast of France or to England, when the period of her delivery approached, in order to escape the scandal. On the night of the 10th of June following, her delivery took place in his house—prematurely, according to the statements of the girl herself, and of Mr. Beasse in his declaration—and in all probability at least unexpectedly, for he had a party of friends with him that evening till midnight, and when they left his house he accompanied them, and did not return home till half past one, although he knew she was unwell. Medical advice was not procured. The girl was delivered about four or five on the morning of the 11th, of a still-born child, as she represented; and in order to keep the affair quiet, and allow her to recruit her strength, she was locked up in her room, and a false story told of her having gone to visit a brother in the neighbourhood. It is particularly worthy of remark that among the steps taken to spread this story, the girl herself, within two hours after her delivery, went to the window of her room, and told her pretended intention to a boy in the garden about forty paces off. About the same time Mr. Beasse went to a friend, the same he had consulted formerly, to ask his advice what he should do; and at this person's recommendation, he allowed a surgeon to visit the girl at mid-day, who found her in bed with the after-birth not expelled, and who examined the body of the infant, without being able to observe any external mark of violence. Mr. Beasse likewise made inquiries as to the best mode of secretly burying the child, but no means could be devised that would satisfy him, by which it might be buried in the church-yard. Various circumstances having excited a suspicion of foul play, the house was visited on the morning of the 14th by a constable, with two medical men. Mr. Beasse admitted that a child had been born; stated that it had either been still-born, or at least when he was summoned to the room by his servant, that he found it lying on the floor amidst a quantity of blood, and respiring feebly; and after several attempts at evasion he conducted the constable to a spot in the garden where he had buried it. The mother, when questioned on the subject, said she was only in her fifth month, that after she was taken in labour she went to bed and there fainted; and that she was ignorant of the sequel till she came to herself, and found the child on the floor dead. She also stated that baby-linen had been provided for the child; but neither she nor Mr. Beasse were able to prove this.

"Both Mr. Beasse and the servant were indicted for child-murder on the 23d of October last. The general evidence brought forward on the trial was nearly the same with what has just been detailed, with the addition of some of the usual contradictions in the declarations of the prisoners, which it appears unnecessary to enumerate. The medical evidence is very minutely reported, as delivered by Messrs. O'Brien and Hoskins, who examined the body, and Mr. Mauger, who visited the girl on the morning of her delivery. All of them agree that the child was not immature, but apparently born at the full time; and Mr. O'Brien adds, in confirmation of this opinion, that the state of the female prisoner on the 14th of June, four days after her delivery, corresponded with that of one who had brought forth a nine months' child. The data in support of these statements are not given, so that we cannot judge of their accuracy. But the opinion founded on the state of the woman is obviously overstretched: at least we question whether any experienced accoucheur would venture to say, from the state of the mother four days after her delivery, whether she had given birth to a seven, eight, or nine months' child.

"The body of the child was buried in a napkin, which was much besmeared with blood and meconium; and some florid blood was oozing from the mouth; but no other sign of violence was visible outwardly. On clearing away the blood from the inside of the mouth, the surgeons discovered a lacerated wound under the tongue, which separated the frænum linguæ a considerable distance backward.—The lower jaw being then detached from the face, the posterior lacerations were found, one on each side of the palate, extending backwards into the throat; the throat, beyond the lacerations was filled with blood, on removing which, a fourth laceration was discovered, wide enough to admit the fore finger, penetrating the upper and back part of the gullet, and laying bare the bodies of some of the upper vertebræ. The cellular tissue between the gullet and windpipe was ecchymosed or infiltered with blood along the whole course of the windpipe down to the root of the lungs. The lungs were high, expanded, and free from putrescence; their colour pale, pink, and mottled; they crepitated when handled, and when cut gave out air and a little blood, they were very buoyant in water, and when a portion was squeezed firmly, it expanded again and still floated. The heart was healthy. The umbilical cord was lacerated about three inches from the navel, and had not been tied. In the abdomen, blood was found besmearing the whole viscera. This proceeded from a wound in the rectum, entering from within about two inches above the anus, passing about two inches more between the coats, and then piercing the peritoneal covering; and the course of the wound between the coats was marked by a line of ecchymosis. This wound appeared to have been made with a sharp instrument. The rectum contained scarcely any meconium; but the rest of the great intestines contained the usual quantity.

"From these facts, both of the medical gentlemen agree that the child was born alive and breathed, and that it had been put to death by means of some instrument introduced into the throat and also into the anus. They deny that it is possible to produce such injuries after death, even immediately after it; they maintain that, in the circumstances of the case, the hydrostatic test, together with the ecchymosis, in all the wounds, is certain proof of the child having been born alive; they are of the opinion that the wounds might occasion speedy death, but that on the whole the wound in the anus was not calculated to produce death with great rapidity, and that those in the throat, though attended with ultimate danger, could not prove immediately fatal except by the concurrence of suffocation, probably produced at the same time by the rude means employed. In particular, however, they express great doubts whether the woman could have inflicted these wounds herself immediately after delivery. In answer to a query as to this point, Dr. O'Brien says 'the word *possible* has so wide a range that I would beg leave not to answer it; but a person in the situation of Sarah Elliot, suffering both mentally and bodily, as she must then necessarily have done, weltering in her blood, and only in part delivered, I would almost say must be more than human to possess the strength of purpose and steadiness of hand necessary to perpetrate at that time the excesses committed on that child.' Mr. Mauger also thinks it almost impossible. Mr. Hoskins waived replying to the query, on the ground that he had not seen the woman before or after her delivery.

"In opposition to this evidence against the prisoner, Mr. Curtis, a surgeon, was examined on the opinions given by Messrs. O'Brien and Hoskins, and states that the child might have died of hæmorrhage from the untied umbilical cord—that it might breathe in the passages before delivery so as to expand the lungs as remarked by the inspectors; that he has known women resume their daily occupations on the very day of their delivery, and has no doubt that some are in such a state as to be able to kill their child—that if a woman, as in the prisoner Elliott's instance, is brought to bed at four or five in the morning, in two hours is able to go to her window and talk to a person forty paces off so as to be heard, and on the same day resumes her daily occupations, and washes her room, she would he able to destroy her child immediately after delivery—that he has actually met with instances of women who immediately after their delivery were so strong that they could have destroyed their children—that the wounds inflicted on the prisoner's child, required little force to inflict them—that ecchymosis may be produced on the dead body.

"Such was the evidence. Both prisoners were found guilty.—Mr. Beasse was found guilty as principal, and sentenced to death, which sentence was subsequently put into execution; and Sarah Elliott, being found guilty as accessory, was sentenced to make the *amende honourable* before the Court, and then to six years' banishment from Guernsey.

"We have but a few remarks to add to this interesting narrative. It is clear the child was born alive. This is proved by the state of the lungs, with the ecchymosis and internal hæmorrhage, which, notwithstanding Mr. Curtis's opinion, could not have occurred after death. It is also clear that the child was put to a violent death, the wounds and hæmorrhage being sufficient to account for it, and such as put accidental death quite out of the question. The only difficulty in the case was, to fix on the actual murderer. One or other of the prisoners certainly was the murderer; for no one else was in the house at the time. But it is by no means so clear to our minds, as it seems to have been to the court, which of the two was the guilty person. Notwithstanding the opinion of Mr. O'Brien and Mr. Mauger, there cannot be a question among well-informed medical jurists, that the female prisoner might have killed the child herself immediately after delivery, as Mr. Curtis very correctly maintained. Such being the case we do not see any evidence to prove who was principal and who accessory; or rather—for the difficulty now stated would in *British* law be no impediment to inflicting the full pains of law on both as murderers—we see no satisfactory proof in the evidence, that there was an accessory at all, and no proof which of the two did the deed. If we suppose that either one or the other murdered the child, there is nothing in the conduct of the remaining party, or in the evidence generally, which might not be perfectly well explained on the principle that he or she acted with the view of keeping quiet what the murderer was endeavouring to pass off as the delivery of a still born child. We are of opinion, then, that the court acted hastily in executing the prisoner Beasse, and feel convinced that he would have escaped either in an English or Scotch court of Justice.—*Ed. Med. and Surg. Jour. April,* 1831.

2. *Can a woman, during the whole course of Utero-gestation, be ignorant that she is Pregnant?*—Most women, accused of infanticide, allege in their defence that they were ignorant of their being pregnant; and some writers on medical jurisprudence admitted its possibility in cases in which the woman has conceived during sleep, in a state of complete drunkenness, or finally in a disease which deprived them of their senses. The motions of the fœtus, however, and other signs of pregnancy must, in most cases, make the woman aware of her condition. M. Orfila nevertheless quotes many cases of married women who had previously had children, and who had no motive for concealing their pregnancy, who went on the full term of their gestation without ever suspecting their condition. Dr. Lozes has related in the *Archives Generales* for February last, the two following cases, which are confirmatory of the opinion of the learned medical jurist just alluded to. Under the head midwifery, Art. 53, p. 521, will be found the opinion of Dr. Elliottson on this subject, and which coincides with that of M. M. Orfila and Lozes.

Case I.—I was consulted, says M. Lozes, in the month of August, 1819, by a female to whom I had before rendered professional services, and who placed the most implicit confidence in me. This woman, taller than common, and of a very thin habit, had the abdomen very much swelled, so much so that she thought herself afflicted with abdominal dropsy. After having examined her with care, I told her that I believed her to be with child: to which she frankly replied that she did not think so, and for the following reasons:—She told me that she was forty-six years of age, that she had ceased to menstruate in her forty-second year, that she had entered the house of a bachelor as a housekeeper, that they had always lived in a connubial state, always adopting *precautions*, (these were her words,) but that for the four years past, when she ceased to menstruate, they thought it unnecessary to adopt these precautions: she also added that she did not experience any of the inconveniences observed by pregnant women, and that she never felt any movements in her abdomen. It is evident from what I have just related, that this woman spoke with frankness, and yet I thought proper to delay for a while prescribing the treatment suitable for the disease under which she believed herself to labour. About six weeks after I was called to this same woman, and brought her to bed of a well-formed child.

Case II.—In the month of October, 1824, being at Rheims, a physician of that city, Dr. Noel, requested me to go with him to visit a lady, who for twenty-four hours past had suffered from violent pains through the whole extent of the abdomen, and which had gone on increasing in spite of diet, semicupia, and emollient fomentations. Dr. N. told me that he believed the uterus to be affected; for, said he, during these six hours past, a sanguinolent sanies has been flowing from the vulva. On our arrival at the patient's, we found her seated, and only suffering from pain at intervals; she informed me that she was fifty-two years of age, that she had been married thirty years, and had her menses regularly until forty-five years old, and always enjoyed good health, but had never borne children.

As there flowed from the vulva, matters tinged with blood, M. Noel solicited the lady to permit me to make an examination, to which she consented, and I own that I was astonished to find the head of a child ready to pass the superior strait.

When I announced that the patient was about to be brought to bed, both she and her husband were not a little astonished; she, who had always been corpulent, assured me that the volume of her abdomen had not been enlarged, and that she had never felt any movements of the child. At all events, I determined, two hours after, to apply the forceps, and this lady was delivered of a living and well-formed child, which she suckled herself.

I ought to add in conclusion, that Dr. Noel had only seen this lady once, from the time in which she had been taken with the supposed colics. It was this which I believe led him into the error.

3. *Medical Statistics of the Royal Maternity Charity.*—During the year 1830, there were delivered in the Eastern District of the Royal Maternity Charity, under the superintendence of Dr. F. H. Ramsbottom,

2221 WOMEN, *of which cases*

27 were twins—about one in every 85 1-2 cases; of these, in 15 cases both heads presented; in 9 the presentation was a head and a breech; in 1, both were a breech; and in 1, a head and a shoulder.

1161 were males.

1086 were females.

2181 were presentations of some part of the head; of which

8 were face presentations—about one in every 273 cases.

59 were presentations of the breech, or some part of the lower extremities—about 1 in every 39 cases.

7 were presentations of the shoulder, or some part of the upper extremities—about 1 in every 321 cases; of these 1 was a second child of twins.

1 was terminated by the process called *spontaneous evolution*; and 1 was complicated, with an adherent placenta.

1 was an entire placentar presentation.

1 was a partial placentar presentation, in which as well as in the entire placentar presentation, it was necessary to deliver by *turning*, as the hæmorrhage did not abate on the membranes being ruptured.

7 were complicated with *dangerous* hæmorrhage before delivery; not the consequence of placentar presentations;—about 1 in every 317 cases. All these cases were delivered naturally, the hæmorrhage ceasing or greatly diminishing on the membranes being ruptured.

20 were complicated with an adherent or retained placenta, requiring removal by the introduction of the hand into the uterus—about 1 in every 111 cases. One of these cases was after a shoulder presentation, and two of them were under twins.

11 were complicated with *dangerous* hæmorrhage after the natural expulsion of the placenta—about 1 in every 202 cases.

1 was delivered by craniotomy, under hydrocephalus.

4 were delivered by the forceps; two by the long—two by the short: about 1 in every 555 cases.

4 were complicated with puerperal convulsions; three before delivery—one after; about 1 in every 555 cases. One of the patients was delivered by *turning*; the other cases terminated naturally.

1 case of apoplexy occurred twenty-four hours after delivery.

In 2 premature labour was induced in consequence of narrow pelvis; about one in 1110. Both the children were born dead. For one of these patients I had induced labour prematurely three times before; the other I had delivered by means of the long forceps twice previously.

1 fœtus was acephalous.
In 1 case there was what is termed a *secondary fœtus*.
14 women died—1 in every 158 1 2 cases.
2168 children were born alive.
79 were still-born—about one in every 28 1-2 cases.

*Of the Deaths.*

1 was on the fourth day after craniotomy had been performed, in consequence of the fœtal head being hydrocephalic. She died from inflammation of the pelvis, and commencing sphacelus in the vagina, induced by the pressure of the head during labour,

2 died from pulmonary disease; one on the sixth day after delivery, from confirmed phthisis; the other a fortnight after delivery, from pneumonia, which commenced towards the close of gestation, was checked for a time, but returned with increased violence after the birth of the child. This woman had a narrow pelvis; she had fourteen childen; on one occasion craniotomy had been performed; premature labour had been induced five times, four times by a gentleman now retired from practice, the last by my father and myself in February, 1829. Her labours had all been difficult, except when brought on prematurely. On this occasion premature labour came on spontaneously, a few days before the time when I had determined to induce it artificially.

1 from a sudden eruption of blood on the ninth day after delivery, under a placentar presentation. Every dangerous symptom had ceased: she appeared recovering favourably; I had discontinued my attendance, when I was summoned hastily, and on my arrival at the house I found her dead.

1 on the sixth day after delivery of twins. I did not see the patient till the day before she died; I was then told that the labour had been natural and easy, but a draining of blood had continued ever since: she was evidently sinking from the loss. On examining the body after death, I found a portion of the placenta attached to the uterus nearly the size of the palm of my hand. Improper attempts to remove the placenta had evidently been made by the midwife.

1 from a ruptured uterus; it was her fourth child; all the children had been born naturally, though dead; her labours had all been lingering, owing to diminished space in the conjugate diameter of the brim of the pelvis. When I saw her first, the membranes had ruptured fourteen hours, and during my absence the uterus ruptured itself. I immediately turned the child, but was obliged to perforate the head before I could extract it.—The rent was transverse in the anterior part of the cervix uteri, and sufficiently large to admit the hand readily into the cavity of the abdomen, into which the whole of the child, except the head, had escaped. She appeared to rally for a few hours; but afterwards gradually sunk, and died on the fourth day after delivery.

1 on the fourth day after delivery by the long forceps: she was much exhausted when the operation was performed, and continued sinking till she died. Her pelvis was considerably smaller at the brim in its conjugate diameter than natural. This was her third child: under all her labours I delivered her by the same instrument.

2 from peritonitis; one on the fourth day, the other a fortnight after delivery. Both labours were natural and easy.

4 from the effects of adherent placentæ. One on the tenth day after delivery, of irritative fever: it was a first child; the labour had been very lingering; the placenta was firmly adherent throughout its whole extent, and removed with great difficulty. The whole was brought away, and there was a very small quantity of blood lost. One was on the ninth day: on the fourth day after delivery she was recovering well; she was afterwards suddenly taken ill, a neighbouring practitioner was sent for, and I did not hear of her again till I was informed of her death.—One on the sixteenth day: she sunk under constant and violent shivering fits, which continued from the third day after delivery till her death. There had been comparatively a small quantity of blood lost, but she was very much depressed by it. The other on the third day, with symptoms exactly such as would be produced by an over-dose of opium. I had ordered her ten minims of laudanum every four hours, and as she had only taken three doses, I at first suspected that the medicine had not been properly prepared. On comparing the remainder however, with another quantity made according to the same prescription, I could not detect the slightest difference in colour, taste, or smell.—Her death may be accounted for in two ways; either the system was peculiarly susceptible of the influence of opium, or, what is more likely, the stupor proceeded from the excessive loss of blood she had sustained; as we sometimes see convulsions the consequence of violent hæmorrhage. The comatose state continued for 15 or 16 hours, and it was with the greatest difficulty she could be roused even to put out her tongue. I never before met with a similar case.

1 of irritative fever, 17 days after the expulsion of putrid twins, at six months; one was expelled two days before the other. There was a slight hæmorrhage after the birth of the second. I removed the placentæ, which were both partly in the vagina, partly retained in utero by the closing of the os uteri upon them. This constriction, and the smallness of the uterine cavity, prevented the introduction of my hand, but both placentæ were extracted whole.

*Of the Still-born Children.*

25 were premature.

9 were putrid at full time, or nearly so.

9 were breech presentations at full time, or nearly so.
4 were shoulder presentations.
1 was under a placentar presentation.
1 was after the mother had suffered from accidental hæmorrhage before delivery.
3 were after the mothers had suffered from puerperal convulsions.
2 were delivered by the long forceps.
1 was hydrocephalic; delivered by craniotomy.
2 were after the induction of premature labour.
1 was under a ruptured uterus.
1 was acephalous.
With 6 the funis prolapsed by the side of the head, and could not be returned. In one of these cases the vagina was much constricted from sloughing after a former lingering labour; I was compelled to divide the stricture by a scalpel.
14 were at full time, head presenting; not putrid, nor delivered by art.

The number of deaths last year was greater than the average proportion in this charity; this may, perhaps, be accounted for in some degree by the prevalence of a puerperal epidemic of a very fatal character, which raged during the commencement of the year in the eastern and north eastern extremities and suburbs of the town. For, although not one of the patients of the Maternity Charity died of that specific disease, still, during its continuance they appeared to suffer more than usual from accidental occurrences, which at other times would probably pass without producing any serious consequences. I particularly remarked that they did not recover so readily after hæmorrhage as I am accustomed to see them, and I suspect that the same condition of the atmosphere which favoured the spreading of the epidemic—without exactly inducing the prevalent disease—predisposed all puerperal women to various unhealthy actions on the application of any exciting cause. —*Lon. Med. Gaz. March*, 1831.

4. *Are there certain Questions which a Medical Man in a Court of Justice may refuse to Answer?*—This is a very important question and one, the solution of which has often been demanded of us. It will appear from the following extracts which we make from a recent No. of the *London Medical Gazette*, that medical persons have *no privilege whatever*, not to disclose circumstances revealed to them professionally; and that the only communications privileged are those to their legal advisers entrusted with those communications as such. A leading case on the aubject is Wilson *v.* Rastall, 4 Ter. Rep. 754.

M. Cæsar Hawkins *examined by* Mr. Dunning. Q. Mr. Hawkins, are you acquainted with the lady at the bar? and how long have you been so?—A. A great many years: I believe about thirty.—Q. Are you acquainted with the present Lord Bristol: and how long have you been so?—I have had the honour of knowing the Earl of Bristol nearly as many years. Q. Do you know of any intercourse between my Lord B. and the lady at the bar?—A. Of an intercourse certainly; of acquaintance undoubtedly. Q. Do you know from the parties of any marriage between them?—*Mr. Hawkins.* I do not know how far any thing that has come before me in a confidential trust in my profession should be disclosed, consistent with my professional honour. (The question and answer were here repeated.) *Mr. Dunning.* I trust your lordships will see nothing in my question that can betray confidential trust, or dishonour to Mr. Hawkins in giving it. My question is simply whether Mr. Hawkins knows from the parties of any marriage between them?—*Lord High Steward.* The question that was asked by the counsel at the bar is, "Whether the witness knew, from any information of either of the two parties, that they were married?" The witness objects to it, whether he is to answer any questions that are inconsistent with his professional honour. Your lordships are to determine whether the question put by the counsel at the bar shall be asked? *Lord Mansfield.* I suppose Mr. Hawkins means to demur to the question upon the ground that it came to his knowledge some way from his being employed as a surgeon for one or both of the parties; and I take for granted, if Mr. Hawkins understands that it is your lordships' opinion, that he has no privilege on that account to excuse himself from giving the answer, that then, under the authority of your lordships' judgment, he will submit to answer it. Therefore, to save your lordships the trouble of an adjournment, if no lord differs in opinion, but thinks that a surgeon has no privilege to avoid giving evidence in a court of justice, but is bound by the law of the land to do it, (if any of your lordships think he has such a privilege, it will be a matter to be debated elsewhere, but) if all your lordships acquiesce, Mr. Hawkins will understand that it is your judgment and opinion, that a surgeon has *no privilege*, when it is a material question, in a civil or criminal cause, to know whether parties were married, or whether a child was born, to say that his introduction to the parties was in the course of his profession, and in that way he came to the knowledge of it. I take for granted that if Mr. Hawkins understands that, it is a satisfaction to him, and a clear justification to all the world. If a surgeon was *voluntarily* to reveal these secrets, to be sure he would be guilty of a breach of honour, and of great indiscretion; but to give that information in a court of justice, which by the law of the land he is bound to do, will never be imputed to him as any indiscretion whatever.

The question was then put once more, and answered directly by Mr. Hawkins.

And *Mr. Justice Buller*, in giving judgment in the case of Wilson *v.* Rastall, above alluded to, thus proceeds:—

"I take the distinction to be now well settled, that the privilege extends to those three enumerated cases, (counsel, solicitor, and attorney,) at all times; but that it is confined to these cases only. There are cases to which it is much to be lamented that the law of privilege is not extended; those in which medical persons are obliged to disclose the information which they acquire by attending in their professional characters. This question was very much considered in the Duchess of Kingston's case, where Mr. Cæsar Hawkins, who had attended the Duchess as a medical person, made the objection himself, but was over-ruled, and compelled to give evidence against the prisoner."

5. *Mode of obtaining the active principle of the Elaterium.*—The great uncertainty of the elaterium as a purgative, probably dependent upon the varying qualities of the drug, renders it of some interest that its active principle should be obtained, so that the uncertainty in the administration of the article should be removed. John D. Morries, Esq. has attained the active principle which he has named *Elaterine*, in the following manner.

Elaterium is to be digested for twenty four hours in distilled water at a temperature of about 200 deg. F. and filtered. The residual matter is to be acted upon by alcohol, s. g. 825; this tincture is to be evaporated to the consistence of thin oil, and while still warm, to be thrown into boiling distilled water; immediately a copious white crystalline precipitate forms, and increases in quantity as the liquor cools. This precipitate is the elaterine, and which is to be separated by decantation and filtration, and repeatedly washed with distilled water. In this state it is sufficiently pure for medical use; if it be required perfectly pure, it is only necessary to repeat the solution in alcohol and precipitation.

The elaterine is a white crystalline substance, of an extremely bitter and rather styptic taste, insoluble in water and the alkalies, soluble in alcohol, ether, and in hot olive oil, sparingly soluble in dilute acids. When in a state of purity, it forms microscopic rhombic prisms, striated on the sides, possessed of considerable lustre, and of a silky appearance when in mass. It is decomposed by the strong acids, forming with nitric a transparent yellowish gummy looking mass, and with sulphuric, a solution of a deep blood red colour. It is fusible at a temperature a little above that of boiling water, and at a higher temperature is dissipated in thick, whitish, pungent vapour, having rather an unpleasant odour.

In the department of materia medica will be found an account of the medical effects of this article.—*Edinburgh Medical and Surgical Journal, April*, 1831.

6. *Process for Economically preparing the Muriate of Morphia.*—Dr. Wm. Gregory, of Edinburgh, recommends the following process for the preparation of the muriate of morphia, without the use of alcohol, and by which this preparation of opium may be made cheaper than any now in use.

"Opium is cut in small pieces, and completely exhausted by cold water, or water at 90 deg. F. The aqueous infusion is concentrated till it occupies a small bulk, and precipitated by a slight excess of ammonia. The precipitate is collected on a filter, washed moderately with cold water, and dried at a temperature below 212 deg. When dry, it is reduced to powder, and rubbed up with cold water. Diluted muriatic acid is now added by degrees. The first portions are speedily neutralized, but fresh acid is added until a slight but permanent excess is present. This dissolves both the morphia and narcotine, forming a dark brown solution, which must be filtered to separate it from some very dark matter which is left undissolved. The filtered solution is now evaporated to nearly the consistence of syrup, and on cooling, forms a brown mass of crystals moistened with a very dark liquid. The whole mass is now subjected to strong pressure between folds of bibulous paper, which absorbs the liquor containing the muriate of narcotine and colouring matter, and leaves the muriate of morphia tolerably pure, although still of a brownish colour. A second solution, crystallization, and expression, yields the salt nearly white and free from narcotine. By a third crystallization the muriate of morphia may be obtained in radiated bunches of silky crystals of snowy whiteness. These crystals, when dried by a moderate heat, become quite opaque. They are soluble to almost any extent in boiling water. Their solution has a very bitter taste, and yields, when supersaturated by ammonia, a highly crystalline precipitate of morphia.—A similarly pure solution of narcotine in muriatic acid gives a curdy precipitate not at all crystalline."

The quantity of muriate of morphine obtained from opium by the above process varies according to the quality of the drug. Dr. G. has however got as much as 17.5 per cent, but the quantity usually procured is about 10 per cent.

Dr. Christian and several other practitioners, who have used the muriate of morphia, consider its powers to be equal to that of any other of the preparations of morphia. It is given in solution, five grains of the salt dissolved in one ounce of warm water, of which twenty-one drops, equal to one fourth of a grain of the salt, is the medium dose.—*Edinburgh Med. and Surg. Journal, April*, 1831.

7. *Fracture of the edgé of the Acetabulum.*—Dr. M'Tyer has given in the *Glasgow Medical Journal* for February last, some interesting particulars respecting four cases of fracture of the edge of the acetabulum, an injury of which little notice is taken by any surgical writer, and yet it would seem not to be of exceedingly rare occurrence, since three of these cases occurred within little more than twelve months in the Glasgow Royal Infirmary. In the first case, a fracture was found to pass through the bottom of the right acetabulum, from below, upwards, and forwards, while a wedge-shaped piece of bone, about an inch and a half long, was separated from the upper and posterior margin of the cotyloid cavity. When the muscles were removed by dissection, this portion of bone was held in place by the capsular ligament and a few fibrous bands at its superior margin, which here formed a kind of hinge. When the head of the femur was brought into its normal situation in the acetabulum, the separated portion of the margin of the acetabulum fell into its proper place. The capsular ligament was lacerated at the lower and posterior part, the tringular ligament was also separated from the bottom of the acetabulum, and there was no resistance offered to the head of the femur raising the detached portion of bone, passing under it, and then gliding on to the dorsum of the ilium, which situation, while the patient was alive, it always assumed, when the thigh was left to the uncontrolled action of the muscles attached to it.

The second case differed from the first, only in this, that the portion of the margin of the acetabulum which was separated, was chiefly from the upper part.

The third case differed from the two former in the situation of the fracture, and there was some dissimilarity in the position ot the limb of the affected side, from that of the two other cases. In this example, as in the first, a fracture passed through the bottom of the acetabulum, but here it was nearly transverse. From that part of the acetabulum opposite the ischiatic notch, a thick, somewhat triangular-shaped piece of bone was separated, removing an inch and a half of its margin; another portion, containing about half an inch of the brim of the acetabulum, was also removed, a little lower than the former, and this adhered to the extremity of that portion of the cotyloid ligament from which the largest piece of detached bone had been entirely separated. The head of the femur was lodged over the ischiatic notch on the pyriformis muscle; the largest portion of bone separated from the ilium was pushed towards the coccyx, whilst the head of the femur had passed under the smaller portion. The capsular ligament was a good deal lacerated at the posterior part; but the anterior, which is much stronger and fibrous, remained entire, and the round ligament was torn across near the bottom of the acetabulum.

The fourth case occurred in a male subject dissected at La Pitie. Nothing unnatural was observed in the position of the limb to indicate that any disease had existed, and it was only when dissecting the pelvis, that more than usual roughness and prominence were observed, occupying the upper half of the posterior margin of the acetabulum; this was at first supposed to be an exctosis of that portion of the ilium, but on more minute examination, Dr. M'T. found it to arise from a portion of bone which had been separated and united with a slight deviation from its natural position; the round ligament was entire, and as the capsular ligament had been in great part removed before Dr. M'T. saw it, he could not ascertain if it presented any appearances to indicate that laceration had at one time been present.

The first case was that of a labourer, aged twenty-seven, on whose back a number of bricks had fallen, while he had his right knee placed on the back of a trench. There was much tense and painful swelling of the right buttock, the right leg was shortened about an inch and a half, bent, and the *toe turned a little outwards.* The limb could be moved without much difficulty, but every motion gave him pain.—When rotated, the trochanter revolved, or seemed to revolve, upon a shorter radius than usual, and a crepitus was distinctly felt when the hand was placed over it. He had also pain in the groin and in the lower part of the abdomen. The pulse was small and the skin cold. On fixing the pelvis, and making extension, the limb could easily be brought to the same length with the opposite, and the deformity at the hip in a great measure disappeared, but the least relaxation immediately reproduced the retraction, and the rounded form of the hip.

In the second case the limb was shorter than the opposite; the knee slightly bent, and turned forwards and inwards, and the toe rested on the tarsus of the opposite foot. The trochanter major was less prominent than natural, and more approximated towards the anterior spinous process of the ilium; there was less flattening of the hip than is generally observed in dislocation. Crepitus was heard, when the limb was not extended.

In the third case the appearance of the limb differed from the first and second, in this, that hardly any shortening was observed, but the toes were stretched downwards, and turned slightly inwards. It was immediately supposed to be a dislocation of the femur; but on attempting to move the limb, very distinct crepitus was heard, and though a good deal of force was used in pulling, we could make but little change in the position of the head of the bone, probably from the rigidity of the muscles, occurring after death.

In the fourth case, it is not known whether any particular symptoms accompanied it when produced; it is not even certain that the head of the bone had been displaced, for the round ligament was found entire; if there was displacement, the head of the femur would have rested on the dorsum of the ilium, and might, in all probability have been mistaken for a fracture of the neck of the femur.

These fractures of the acetabulum, it is evident, may, on even a pretty accurate examination, be mistaken for a fracture of the neck of the femur, or a dislocation, and though there is no symptom which by itself will indicate either of these injuries, yet a combination of symptoms will pretty clearly point out the one from the other.

From the appearances which the cases presented previous to dissection, a different injury from the true one was suspected. The first case was taken for a fracture of the neck of the femur, and the symptoms differed in no respect from those set down by writers on the subject. There was the diminished length of the limb—the crepitus on rotation—the facility of extension and reduction. The only symptom which rendered the case doubtful was the ultimate turning inwards of the toes, and even that has been given as a symptom usually accompanying fracture of the neck of the femur by Pare, Petit, and B. Bell. Desault has also mentioned cases which he says were cured without any shortening of the limb, where this symptom was present; and it is not denied by any writer that it may occasionally occur. The second case was in like manner mistaken, and the third was supposed to be a dislocation, from the appearances which the subject presented, though the crepitus rendered this doubtful. The limb was slightly shortened, and the toes turned downwards and inwards, and the limb could not be drawn into its natural position. It would probably have been different had the patient been alive, for this immobility in all probability arose from rigidity of the muscles occurring after death.

That combination of symptoms which indicates fractures of the edge of the acetabulum with displacement of the femur, of which all are not present either in fracture of the neck of the femur, or dislocation of the femur on the dorsum of the ilium or ischiatic notch, is the following:

The limb of the injured side assumes the position of one of the two dislocations mentioned. There is a crepitus previous to extension being made. The reduction of the limb to its natural position is easy, but there is difficulty in retaining it there. If firm pressure is applied over the buttock after extension has been made, no crepitus is observed. There is less flattening of the buttock than in dislocation.—There is increased mobility of the limb after extension, and the pain experienced on rotation of the limb being made after reduction, is less than it was before that. They differ from dislocations without fracture of the acetabulum, therefore, in the presence of crepitus—in the facility of reduction and the immediate return of the femur to its unnatural position, when left to the uninterrupted action of its own muscles.—They differ from fracture of the neck of the femur, in the position of the limb, the toes being turned inwards, and in the presence of crepitus before extension has been made.

Dr. M'T. is of opinion that in some of those cases of reported cure of fracture of the neck of the femur within the capsule, where no autopsy was obtained, and especially such as presented amongst the symptoms the toes turned inwards, there was probably fracture of the edge of the acetabulum, while the femur was entire.

8. *Fracture of the body of the Scapula.*—James Syme, Esq., relates in the *Edinburgh Medical and Surgical Journal* for April last, a case of this rare accident. It occurred in a man aged forty-five, who, whilst carrying a heavy stone in a handbarrow, across the sunk area of a house, the wooden gangway broke, and he was precipitated to the bottom. He fell first, and the stone struck him on the back. The lower portion of the scapula was drawn upwards, and projected by the action of the *teretes* muscles, together with the *latissimus dorsi.*—Mr. S. put a cushion of tow under the axilla, another over the lower part of the scapula, and then applied a spica bandage, under which treatment the patient felt quite easy, and was dismissed free from uneasiness or deformity in three weeks.

9. *Dislocation of the Sacro-iliac Symphysis.*—An interesting case of this rare accident is related by T. E. Baker, Esq., in the fourth volume of the *Transactions of the Medical and Physical Society of Calcutta.* The subject of the case was a lieutenant of cavalry, a muscular man, aged thirty-six. While on parade his horse stumbled and fell; the rider "was thrown forward over the horse's head, and was on his knees and hands, when the horse, in recovering his fall, again fell, struck him on the perinæum with his head, and came with the whole weight of his body upon the left hip; to use his own words, 'the horse appeared to drive him into the ground, he heard the bones make a noise like the rattling of a bag of pebbles, and he thought his bowels were driven out in front; he found he could not stand, and when being placed in the *dooly*, the bones made a snap, and gave him great pain.'

"When placed on his right side, a slight projection was perceptible at the posterior and superior spine of the left ilium; but when lying on his face, the projection was not perceptible, nor was there any pain on pressing the part, nor any appearance of fracture. On turning him again on his right side, pressing on the hip joint, and bending the thigh towards the abdomen, the bones of the pelvis made a harsh grating noise, and the motion gave great pain. On being turned on his back he was easy except a slight pain at the sacro-iliac symphysis.

"At 11 A. M. he was bled to about 24 ounces, when he nearly fainted; his bowels had been opened early in the morning, and he made water only a few minutes before the accident occurred; his pulse was natural, and 76; he had great pain in the urethra in passing his urine, which was tinged with blood; in the afternoon he complained of pain in the scrotum, in the left groin, and over the pubes, to which thirty leeches were applied; in the evening he complained of pain, and numbness in the left groin and thigh, to which warm fomentations were applied; he took light nourishment during the day, was in good spirits, and wrote a note to his brother and myself. We applied a linen roller round the body, made a hole in the bed and bedding, and contrived a sort of leather funnel, so that he should have no occasion to move when the bowels were relieved.

"18th.—Passed a tolerable night, having slept several hours; complains of great pain in making water, which is still tinged with blood; he once heard the bones crepitate when moving unconsciously, but it did not give him much pain; pulse 74 and natural, tongue clean, and moist; has pain higher up in front of the abdomen; above the pubes; bowels not being moved, he took a table-spoonful of castor oil, which having no effect, was repeated at 2 P. M.; applied a soft leather belt around the body instead of the linen roller, but he cannot bear the lower straps to be tightened, as the pressure gives him pain above the pubes.

"19th.—Was restless during the night, and is much worse this morning, has vomited several times, and is much distressed with hiccough; the urine dribbles away guttatim and gives him much pain. Effusion has taken place into the scrotum and perinæum, but to no great extent. We tried to pass the catheter, but could not succeed, either from the urethra being ruptured, or from a stricture of some years' standing; pulse 130, weak and low; cold perspirations on the forehead; has had several loose stools. The hiccough is much relieved by taking ether with camphor mixture, which he says he finds very refreshing.

"20th.—Was very restless in the early part of the night; had two stools; passes more urine and with much less pain; hands quite cold; pulse 145 and very weak; the effusion into the scrotum does not increase, and it has assumed a livid appearance; tongue is clean and moist, but he complains of being very thirsty; has been reading during the morning, is quite sensible, and free from pain, and can scarcely believe that he is in great danger, though he is evidently and rapidly sinking; since the morning the whole of the abdomen is swollen very much, and appears to be distended with air: he is continually dozing, but being unable to obtain sound sleep, at 10 P. M. I gave him, at his own request, thirty drops of laudanum, but he rejected it almost immediately. The hiccough still continued, but he remained free from pain, and died at 2 A. M. the following morning, (the 21st.)

"The body was examined ten hours after death; the cavity of the abdomen and its contents had a livid, purplish appearance, as if blood had been extravasated through the whole extent of the peritoneum; it contained about six ounces of blood, on which a small quantity of oil floated; the stomach exhibited effused blood on its external surface, and several clots were observed on its inner membrane. The liver had numerous and firm adhesions to the diaphragm; there was dark discoloration on its right lobe, and on raising it, a quantity of thick, florid, coagulated blood was found underneath. The intestines were much distended with air, and in several places had the appearance of being bruised; there was a very small opening in the colon less than the size of a pea; we were not certain whether it was ruptured, or whether it was caused by the knife; when the intestines had been exposed to the air for about half an hour, they turned of a greenish hue; on removing them, and detaching the psoas and iliac muscles from the os innominatum, on which there was much effused blood, a total separation of the ilium from the sacrum was discovered, at the sacro-iliac symphysis, and a small transverse fracture of the ilium, nearly two inches in length. On cutting through the recti muscles over the pubes, about an ounce of brown coloured fluid escaped, of a urinous smell. The ossa pubes were disjointed at the symphysis, the cartilage was torn from the bone on the left side, leaving the surface rough, and there was a space of about half an inch between them. The bladder contained a small quantity of urine, and its external and internal surface had a dull red appearance. The urethra was ruptured, just before its passage through the triangular ligament, and some urine had escaped into the adjacent cellular membrane; the stricture was about six inches from the orifice of the urethra.

"From the very great injury the parts had sustained, it may appear surprising that the patient suffered so little pain; for, excepting when passing his urine, he was quite easy."

A case of this variety of dislocation is related in the memoirs of the Academy of Dijon; and another, which occurred to Dr. Harris, will be found in the fourteenth volume of the Philadelphia Journal of the Medical and Physical Sciences.—*American Journal of the Medical Sciences.*

10. *Perforation of the Membrana Tympani.*—It has been believed that in certain cases of deafness, advantage has been obtained from perforating the membrana tympani, but as the wound almost always soon heals, the benefit has been but temporary; and some ill consequences have sometimes resulted from the inflammation which follows the operation. Dr. SOLERA, in a communication published in the *Annali Universali di Med.* for Jan. last, states that he is satisfied that these ill consequences are rather attributable to imperfection of the mode of operating, than to the operation itself, and he recommends it to be performed by introducing a piece of gum elastic catheter into the external meatus down to the tympanum, and through this, to pass a probe armed with caustic potash. When the potash touches the tympanum, a rotatory motion is to be given to it. The loss of substance thus occasioned, prevents the future obliteration of the opening.—*Ib.*

11. *Wounds of the Throat.*—Baron LARREY, in his *Clinique Chirurgicale*, relates some interesting cases of this description: we give the following abstract of them from our esteemed cotemporary, the *Medico Chirurgical Review.*

CASE I.—M. Arighi, (now Duke of Padua, and then aid-de-camp to General Berthier,) received a musket ball in his neck at the siege of Acre, by which the external carotid artery was cut across, near to the place where it is given off from the internal, and as it enters the parotid gland. The gush of blood from both apertures of the wound attracted the attention of the artillerymen, and one of them instantly pushed a finger into each opening, and thus arrested the flow of blood. Baron Larrey was immediately called amidst a shower of shot and shells. He applied pressure and maintained it carefully for some days, by which means, and without any ligature, life was preserved, and all hæmorrhage prevented.

CASE II.—After the battle of Waterloo, the baron had an opportunity of seeing a young English soldier who had the left external carotid artery *partially* opened. The hæmorrhage was alarming; but the English surgeon cut down on the aperture, and tied the artery both below and above the wound. The patient entirely recovered.

CASE III.—Henry Cabon, of the Swiss Guard, was brought into the Hospital de la Garde, on the 21st of November, 1828, immediately after receiving a sabre-wound, while fighting a duel, in the upper part and right side of the neck. When the baron arrived, the man was nearly dead from hæmorrhage and suffocation. The wound was laid bare, while an assistant made pressure on the line of the artery, and then the baron enlarged the orifice, and discovered that the superior thyroid artery was wounded, as also the external carotid itself. A cellular pouch had formed behind the thyroid gland, (which was goitrous,) filled with clotted blood, and which was pressing on the trachea. The removal of these clots was followed by a jet of arterial blood.—

The baron was unable to seize the vessels from which the blood issued, and therefore laid bare the trunk of the common carotid, and passed a ligature around it. He was not a little surprised to find this artery no larger than the radial artery at the wrist. This was attributable to the great loss of blood. The great source of hæmorrhage was thus cut off; but some vessel still continued to supply blood at the upper part of the wound. This vessel was fortunately seized by the forceps and secured. The wound was then cleaned and dressed, the breathing continued difficult, and the lips deadly pale. For two or three days it was doubtful whether this man would rally, but eventually he recovered.

Case IV.—A grenadier of the army of Egypt was wounded by a bayonet, the broken point of which remained, for six weeks, deep in the side of the pharynx, behind the arch of the palate. The man had entirely lost his voice. The baron, with great difficulty, seized the foreign body and extracted it. The voice was instantly restored.—The iron had pressed on the laryngeal branch of the par vagum.

Case V.—A subaltern officer of the guards was brought into the hospital on the 7th of June, 1824, presenting a wound in the neck, on the right of the larynx, so small as to be scarcely perceptible. There was great ecchymosis and tumefaction of the whole anterior region of the neck, with deep-seated pain in the chest. Voice and speech were gone—the respiration exceedingly difficult, as well as deglutition. He informed Baron Larrey, by writing, that this wound was made by a small sword. Venesection was repeatedly employed, together with cupping and leechings, which gave some relief. On the sixth day, however, he was menaced with suffocation, and his face was blue and bloated. The baron found him apparently in the agonies of death.—In this crisis he determined on tracheotomy. He made an incision through the integuments of some length, and then perforated the space between the thyroid and cricoid cartilages. An immense explosion of air was the immediate consequence, together with the expulsion of several clots of blood. Respiration succeeded, and considerable relief was the result. A paroxysm of suffocation, however, soon after occurred, owing to the obstruction of the orifice in the air-passage, and a tube was quickly inserted. Relief was again obtained; but thirst was intolerable, and the unhappy patient was unable to swallow. In this dilemma, a tube was with great difficulty passed into the stomach, and fluids introduced into that organ. The thirst was moderated, but he could not bear the presence of the hollow bougie, and tore it out himself. He lingered in dreadful agony, till 4 o'clock the next morning, when he expired.

On dissection, an abscess was found in front of the three superior cervical vertebræ, (which were denuded,) the size of a hen's egg, and which had pressed so much the parietes of the pharynx against the cricoid cartilage and upper part of the trachea, that respiration could not be carried on through the aperture that was made by the knife. A purulent infiltration had also penetrated down into the chest through the cellular membrane.

The baron, in his remarks on this case, does not allude to the possibility of life being saved, if the opening had been made lower down in the trachea, instead of the place which he pitched on for the operation. In all cases where tracheotomy is deemed necessary, the lower down the operation is performed, the more difficult it is—but the greater is the chance of success, for the obvious reason that we are thus more likely to get below the obstruction.

Case VI.—General Murat, (afterwards king of Naples,) received at the battle of Aboukir, a musket-shot, which traversed the neck from side to side, wounded the root of the tongue, and carried away a portion of the epiglottis. The baron was on the spot, and rendered immediate assistance. The first phenomenon which he observed, was the discharge of the dismembered portion of the epiglottis, followed by a considerable expectoration of frothy blood. The general was harassed for some days with painful cough, loss of voice, &c. The baron cleared the orifices of the wound both at its entrance and exit, and then introduced an elastic tube in the œsophagus, for the purpose of introducing liquid nourishment and drink into the stomach. This was necessary, as there was no proper valve to prevent the ingress of substances into the trachea. In the course of eighteen days, however, the parts had so accommodated themselves to the loss of a portion of the epiglottis, that this officer was able to swallow with little or no inconvenience.

Case VII.—In this case, which was that of a soldier in Egypt, who was wounded by a musket-ball on the 21st of March, 1801, the whole of the epiglottis was carried away. The poor fellow was devoured by thirst, but could not drink, and harassed with incessant cough. In this dreadful state he continued four days, without any relief. When Baron Larrey saw him, he was in the most piteous and dangerous condition. The baron was enabled to pass a gum-elastic tube down the œsophagus, and through this to introduce liquids into the stomach. By a long and assiduous perseverance in this measure, the life of the soldier was saved, and nature supplied the place of the epiglottis by a contrivance of her own.—*Ib.*

12. *Tracheotomy.*—The expediency of opening the trachea in those instances in which a foreign body is lodged in one of the bronchial tubes, does not appear to be as yet entirely decided by surgeons. To aid in settling this interesting question, we have been careful to lay before our readers from time to time, such cases as appeared to throw any light upon the subject. An interesting memoir has been recently read to the Dublin Chirurgical Society, by John Brown, M. D. and which, consisting principally of an analysis of cases, does not admit of condensation within the limits to which we are here restricted, and we must therefore content ourselves with the conclusions he has drawn from them, and which appear to us to be legitimate inferences.

"1. That the existence of foreign bodies in one or other *bronchus* can be ascertained by the use of the stethoscope; by the seat of the pain and other uneasy sensations; and by the previous history of the case.

"2. That since the effect of such bodies in these unnatural situations, is to excite inflammation and abscess, most commonly ending, sooner or later, in death, it is incumbent on us to attempt their extraction with the least possible delay.

"3. That small round bodies move freely from the *bronchi* to the trachea, particularly when an opening has been made in the latter, and that the best mode of promoting their expulsion is by such an operation.

"4. That when sharp and angular substances have descended into either *bronchus*, they generally become fixed there, but may be extracted by forceps or other suitable instruments passed through an artificial opening in the trachea.

"5. That the sooner such an operation is undertaken, the greater will be the chances of success, as the presence of the extraneous substance must give rise to congestion and inflammation in the lungs, and to various cerebral affections, all depending on mechanical interruption to the natural course of the circulation.

"6. That although occasional recoveries have ensued subsequent to the spontaneous ejection of foreign bodies from the *bronchi*, such cases are rare; and the great number of persons so circumstanced have died at longer or shorter intervals."—*Ib.*

13. *Idiopathic Glossitis.*—In our first vol. pp. 213, 219, 448, and in vol. four, p. 533, will be found accounts of four cases of glossitis; the following cases, related in the *Glasgow Medical Journal*, for February last, by JOHN ORGILL, Esq., are interesting, as contributions to the history of this rare affection.

CASE 1.—C. Kenmuire, a farmer, aged 50, complained of much difficulty in deglutition, which he attributed to inflammation of the throat. As he lived at some distance in the country, and could not come in himself, his wife came to me and explained the symptoms as well as she could. Appropriate remedies for the supposed disease—inflammation of the throat—were recommended. About a week after this, he was brought to town in a cart. I then found that the left half of the tongue was so much swollen, as completely to prevent articulation and deglutition. The right half of its natural size and appearance, was in part overlapped by the diseased half. For eight days preceding, he had not been able to swallow any thing solid; and during the last two days, he could not get down a drop of liquids. The pulse was nearly natural. I wished to apply leeches to the tongue; but the mouth was so completely filled with it, as not to afford space for them, except at the tip. I therefore put eleven large leeches to the root of the tongue externally; and, when they fell off, applied a cupping glass over the bites, by which means about six ounces of blood were obtained. But this afforded little relief. I then introduced a scalpel flat on the dorsum of the tongue; and made two incisions about half an inch deep, from the furthest point to which the instruments reached to the tip.

The incisions bled pretty freely; and the swelling was, in consequence, so far reduced as to enable him to answer questions intelligibly. He could also expectorate a little, which he was before unable to do, though, as he expressed it, "choking with his spittle," which was thick and very tenacious.

This was at noon. I saw him again about eight o'clock in the evening. The diseased half of the organ was then as much swollen as ever. I scarified it still more deeply; and ordered an enema with an ounce of castor oil. As he was evidently exhausted from want of food, for which he had a good appetite, but which, as I have stated, he had been unable to take for eight days, I ordered some soup to be made, with the intention of calling in an hour after, and of attempting to introduce it into the stomach by means of the stomach-pump. I accordingly returned, and found him smoking his pipe, the last scarification, along with the enema, having given him great relief. With considerable difficulty, and very slowly, he swallowed a small bowl of soup. When I saw him next morning, the tongue had resumed its former swollen state. I then observed, what I had not done before, a peculiar lividity at the tip of the diseased half of the organ. I now introduced the scalpel as before, and made an incision more than an inch in depth. A great gush of most offensive pus followed, and gave the patient immediate relief. The incisions healed in eight days; the tongue having recovered its proper size and appearance. The sensibility on the left side of the tongue continued impaired for a year after, but it was afterwards gradually recovered.

Case 2.—March 5th, 1828, James Brown, a sailor, æt. 35, after languor, and some rigour, complained of difficulty of deglutition, which he attributed to inflammation of throat. I saw him next day, and found the left half of the tongue swollen to at least three times its natural bulk, and very painful. Articulation and deglutition were performed with much difficulty and pain. The surface of the tongue was foul, except at the tip, which was peculiarly clean and red.—The papillæ of this part seemed to have entirely disappeared, leaving the tip remarkably smooth. The median line formed an abrupt termination of the enlargement. Pulse 100, very hard and full. Some thirst. The abstraction of twenty ounces of blood gave some relief, and enabled him to swallow a brisk cathartic immediately after.—The next day the tumefaction and pain seemed to be again on the increase. Five large leeches were applied to the tongue; and the cathartic was repeated. The leeching gave immediate relief; and, from this time, the disease rapidly abated, leaving the organ in a healthy state on the fourth day after the attack.

Case 3.—July, 1828. J. B., a woman from the country, applied to me with glossitis affecting the whole organ, and terminating in suppuration of the right half. She was relieved by scarifications, by letting out the pus, by the lancet introduced at the side of the tongue, and by cathartics. Some months after, she was again attacked with the same complaint. As I was hurriedly called away when she came to me, she went to another surgeon, and I never learned the result. In this case, likewise, there was a peculiar lividity, and smoothness at the tip, on the side which suppurated.

*Remarks.*—In none of these cases could the patient assign an adequate cause for the complaint, unless we consider as such, the only one that Kenmuire could give. At the first bite of a very sour apple, which he had been eating two days before the attack, he felt as if a needle had run into his tongue; and a sudden flow of saliva followed. In the 4th vol. of the Dublin Hospital Reports, a case of idiopathic glossitis affecting the left half of the tongue is related by Dr. Graves.

and is, apparently, the only case on record in which inflammation was limited to the half of the organ. In the first two cases related above, the disease was confined to the left half also. This, of course, must be considered as an accidental coincidence; for we can hardly conceive why the left half should be more liable to inflammation than the right. Perhaps the lividity on the tip in the first and last cases may be considered as symptomatic of the suppuration which took place. If so, this would encourage us, in a similar case, to have recourse to incision as practiced in these cases with so much success. I believe it will be found very difficult to detect the presence of pus by the feeling of fluctuation which generally guides us in other cases. The tongue fills the mouth so completely, and the introduction of the fingers gives so much pain, that putting out of the question the unsteadiness of the organ, its peculiar texture, and the deep seat of the pus, it may be considered a matter of some importance to fix on some appearance as indicative of the formation of an abscess. So far as these cases go, the livid colour of the tip of the tongue may be considered as symptomatic of suppuration.

14. *On Permanent Involuntary Contraction of the Muscles.* By Samuel Smith, Esq. of Leeds.—It is not uncommon in surgical practice to meet with cases, where certain muscles have remained for a great length of time, rigidly and permanently contracted. This state sometimes results from disease in the nerve distributed to the affected muscles; occasionally it is produced by the muscles having their points of attachment unduly and unnaturally approximated for a considerable length of time—as in unreduced dislocations—in the treatment of fracture, &c.; and, in some cases, the precise cause cannot be ascertained.

When a muscle has long been in this state, it often remains contracted, solely from habit, even after the cause which originally produced it has ceased to operate; and by breaking this habit, relief may generally in a short time be obtained.

There are certain sets of muscles which act as antagonists to each other, as for example the flexors and extensors of the arm. The contraction of either of these sets of muscles is always accompanied with a simultaneous relaxation of the other. Thus, if the arm be powerfully flexed by the biceps, and the extensors brought into action, the extensors no sooner act than the biceps become relaxed.

Suppose then the flexors of the arm to have been some time in a state of permanent involuntary contraction; if the limb, by gentle force, be put in the position of perfect extension, the flexors become relaxed, and by maintaining this position a certain length of time, this unnatural *habit of involuntary contraction* which has been acquired in the flexors, may be broken or destroyed. To prove the success which may be expected to follow this plan of treatment, the following cases are selected from many others which have come under my notice.

Mary Leak, aged 25, a stout, robust woman from the country, was admitted a patient of the Infirmary under my care, July 30, 1820.—

She had been fifteen months under treatment, suffering much during the whole of this time from permanent contraction of the quadriceps extensor femoris, the whole of which muscle was in an extremely rigid state. She walked without pain, but an inability to bend the right knee in the least degree gave her the appearance of having a wooden leg. The warm bath, frictions, and many other means had been persevered in for a great length of time, without producing the least effect upon her complaint. On the day succeeding her admission, I placed her on the bed on her left side, and taking hold of the ankle with my right hand, grasping the thigh with my left, I succeeded in drawing the heel and pressing it against the buttock, thus producing a perfect flexion of the limb. It is necessary to explain that in accomplishing this, recourse was had more to art and cunning, than to force. It was gratifying to find that the rigid muscles had become perfectly relaxed, and in order to destroy the tendency to reaction, two leather straps with buckles were placed tight round the upper part of the thigh and ankle, binding the limb in this position, the heel touching the buttock.

She was ordered to remain in bed bound in this manner until my visit on the following day. The relief was immediate and complete. Upon being released next day, it was found that the muscles, which had been for so long a period contracted, were quite relaxed; and not only so, but the tendency to involuntary contraction was destroyed. Suspecting, however, it might return, she remained an in-patient ten days: no return of the complaint took place; she was made an out-patient, and appeared as such August 30. She was perfectly well, and had suffered no relapse.

October 20, 1826. William Holdin, aged 36, admitted a patient of the Infirmary, under my care, on account of the right masseter muscle being permanently contracted. He has been fourteen months incapable of opening his mouth more than to admit the handle of a leaden spoon. Upon introducing the finger within the cheek, and the thumb without, the muscle can be grasped, and in hardness it resembles bone rather than muscle. He has been upwards of a year unable to close the right eye. He was directed to wear a wooden wedge between the teeth so as gradually to open the mouth, and thus gain upon the contracted muscle. No medical treatment was adopted, and in the course of a week or ten days the mouth could be opened upwards of an inch; the masseter muscle had become relaxed and soft, and he was so much relieved that at his own particular wish he went out, November 10th, in order that he might labour for his family; he was, however, directed to continue the use of the wooden wedge for some time. He was able to take common diet, which had materially improved his strength, having previously lived a long time upon spoonmeat, from his inability to open the mouth: he could also close the eye, which he had not done for upwards of a year.

November 2d, 1829. Miss H., a young lady, residing about twenty miles from Leeds, had the misfortune, nine weeks ago, to fall and sprain her wrist, for the relief of which, leeches and the usual applications were had recourse to, under the direction of a very respectable pracwere had recourse to, under the direction of a very respectable practitioner; in a few days she was better of the sprain, but the ring and little finger were permanently contracted, and she had lost the power of extending them: to relieve this affection, various means were had recourse to without effect; she then came to Leeds to place herself under my care. Finding she had considerable pain upon pressure, in the course of the ulnar nerve, I thought it advisable previous to extending the fingers, to apply a small blister, (three inches long and one broad,) above the wrist, and in the direction of the nerve. The day following, the fingers were greatly extended; dressings applied to the blisters, a compress of lint, and a splint reaching from the extremity of the fingers a little beyond the wrist, was firmly secured by a bandage to keep them extended.

Next day they were removed, the contraction of the flexors had ceased, she had the perfect use of her hand, and has suffered no relapse up to the present time, (June, 1830.)—*North of England Medical and Surgical Journal for Nov.* 1830.

15. *Absorption of the Iris.*—Mr. Middlemore, assistant surgeon to the Birmingham Eye Infirmary, states that he has several times known laceration of the iris from local injury, followed by its partial, and in one instance, its total absorption; and in every case that the organ so injured, has, eventually, become amaurotic.

A boy, he says, received a blow upon the eye, from a piece of metal, which was followed by considerable pain and inflammation, and laceration of the iris; for a time, the pupil was cordiform, being pointed at its lower part; slowly and without any pain, the whole of the iris was absorbed, and the eye became amaurotic.—*The Midland Medical and Surgical Reporter, Feb.*, 1831.

16. *New operation for the cure of Ptosis.*—R. T. Hunt, Esq., assistant surgeon to the Manchester Institution for curing diseases of the eye, has proposed in cases of ptosis, occasioned by the loss of power in the levator, whether attributable to the actual destruction of a part of the muscle, or to paralysis of the nerves supplying it, caused by injury or disease, an ingenious mode of treatment, by which the action of the occipito-frontalis is substituted for that of the levator. In the action of raising the eye, the levator palpebræ is not the only muscle brought into play. The anterior portion of the occipito-frontalis also considerably contributes to this effect, by elevating the superciliary integuments into which it is inserted. These muscles, says Mr. H., act so much in concert, that it is almost impossible to draw the eyebrow upwards, whilst the eye remains perfectly shut, and equally difficult to depress the eyebrow, whilst the eye remains wide open. And when we reflect that the origin of the levator is situated at the very extremity of the orbit, and that it is inserted into the tarsus, a part so easily moved, it becomes evident that, unless this muscle's action were restrained by some other power, the tarsal margin would be drawn too far into the orbit. The anterior fibres of the occipito-frontalis which are so inserted into the superciliary integument, as when in action, to stretch the upper part of the skin of the eyelid, constitute this power; and it is with reference to this combination of muscular actions, that the following method of operating is recommended.

The operation is performed by dissecting off a fold of integument; the difference from the usual way of proceeding consists in the portion removed. The upper incision is made immediately below the line of hairs forming the eyebrow, and extends each way, to a point, opposite the commissures of the eyelids. In making the lower incision, no precise direction can be given. It should approach within a short distance of the tarsal margin, varying in the extent of the portion included between the two incisions, according to the greater or less degree of relaxation of the skin, which is different in any two individuals, and it should meet the upper incision at both extremities. When the intervening portion has been detached, the divided edges should be accurately united by, at least, three sutures, and the wound dressed in the usual manner.

The effect produced, when adhesion is perfected, is the attachment of the eyelid to that portion of the skin of the eyebrow, upon which the occipito-frontalis acts, and by means of this attachment, substituting the action of this muscle in raising the eyelid, for that of the levator, which is no longer capable of doing so.

Mr. H. states, that the removal of so large a portion of skin does not produce, contrary to what might be supposed, any deformity.

17. *On the Energetic Contractions of the Heart as a guide in the employment of Venesection.*—It has long been acknowledged that the pulse cannot always be considered as a sure guide in the employment of venesection; an operation which is often necessary even when the state of the pulse seems to contraindicate its employment. "In cases of this sort," says Laennec, "the stethoscope affords a rule much surer than the pulse: whenever the contraction of the ventricles is energetic, we may bleed without fear; the pulse will rise. But if the contractions of the heart are feeble, although the pulse still retains a certain degree of strength, we must be cautious respecting the employment of venesection."

Dr. Graves, in a late clinical lecture, delivered at Sir Patrick Dun's Hospital, Dublin, and a report of which is given in the *London Medical Gazette*, for March last, stated, that for aught he knew, the latter proposition might be correct, but he decidedly denied the truth of the assertion, that whenever the contraction of the ventricles is energetic, we may bleed without fear; an assertion, he says, so much at variance with the results of his experience, that he rather thinks it must have been the offspring, not of observation, but of theory. "In the first place," says Dr. G. "in persons who have hypertrophy and dilatation of the heart, the ventricular contractions will be energetic often until a short period of their death; and under circumstances in which venesection would be totally inadmissible. Of this I have lately witnessed two examples, and one of them made a deep impression upon my mind, as, misled by the precept of Laennec, I was induced to bleed the patient, attending only to the violence of the heart's pulsations, and not to the general symptoms. In this case, the man died in the course of a few hours after the venesection. Dissection, indeed, revealed lesions which proved that death was inevitable under any mode of treatment; but still that event was no doubt accelerated by the bleeding. Nor do diseases of the heart form the only exceptions to Laennec's rule; for I can asert, in the most positive manner, that I have seen cases of pneumonia in which the heart's pulsations continued violent until within a short time of dissolution: so much so indeed, as to induce the erroneous belief in myself and other medical attendants, that this organ was in a state of hypertrophy and dilatation; and yet, it was found, after death, to be in every respect healthy. In these cases, the extent of pulmonary hepatization which was detected on dissection, together with the shortness of the interval which elapsed between the time when we observed the heart's pulsations to have been so violent, and the fatal termination of the disease, made us rejoice that no further use of the lancet had been made; as the patients' friends would have most probably attributed death to the untimely loss of blood. I am the more anxious, gentlemen, to impress upon you these facts, because the language in which Laennec expresses himself upon this subject is so strong. His concluding observation is as follows. "The certainty and facility with which the cylinder indicates the propriety of blood-letting in such cases as those above-mentioned, (which have been hitherto considered the most difficult in practical medicine,) appears to me to be one of the greatest advantages to be derived from the employment of this instrument. It is certainly of the most general application, as it refers to the employment of one of our therapeutic measures, which is the most ingenious or the most useful of any, and which may be had recourse to in almost all diseases."

It is with regret I find myself obliged to differ, on this practical point, from one to whom our science perhaps owes more than to any author who ever lived.—*American Journal of the Medical Sciences, August*, 1831.

18. *On the Treatment of Hooping Cough.*—By Dr. A. T. Thomson, of the London University Dispensary.—In the treatment of hooping cough, Cullen's method of dividing the disease into three distinct classes is highly judicious; each stage being marked with its characteristic features, which distinguish it from the others, and each requiring a distinct plan of treatment.

In the *first stage*, the treatment must be regulated by the type of the fever. Thus, although bleeding is proper when the excitement is considerable, or when the pulse is hard, small and quick, and the breathing oppressed, indicating congestion, yet, as it is an acknowledged maxim that the symptoms of spasmodic affections are rather increased than diminished by the use of the lancet, it would be highly injudicious to bleed, unless such symptoms as have been just described clearly point out the necessity of abstracting a portion of the circulating mass. In my own practice, I have seldom had occasion to employ bloodletting; but in some of the cases in which it has been requisite, the necessity was not limited to the commencement of the disease; and in one distressing case for which I was consulted some weeks since, bleeding, although it did not save the patient, yet greatly soothed his sufferings, and rendered the closing hours of life comparatively comfortable.

Nothing is so likely to produce that state which requires the use of the lancet in hooping cough, as the imprudent exposure of the patient to sudden alterations of temperature; and therefore, when we consider also how much milder the disease is in warm climates, the propriety of confining those laboring under it to one or two apartments, kept at a medium temperature, must be obvious. The custom of frequently changing the air was founded on an erroneous view of the disease, and is now, I believe, rarely recommended, until the disease has nearly run its course, when the change, operating as a tonic power, rapidly annihilates the cough, which sometimes continues, from force of habit, in persons of weakened and irritable constitutions.

The early employment and repetition of emetics in hooping cough, is a judicious remnant of the old humeral practice in this stage of the disease. In infants, emetics free the stomach not only of the mucus from the trachea and bronchia, which is brought up in coughing and immediately swallowed by very young patients, but also, in those more advanced in years; they dislodge from it that preternatural secretion of mucus, which has been already mentioned as occurring in this organ; for it is a well-known fact, that, through the connexion of the stomach and the larynx by means of the par vagum, nauseating medicines, rousing the stomach to discharge its contents by vomiting, loosen the adhesion of viscid mucus to the lining membrane of the larynx and trachea. Emetics also determine powerfully to the surface; and when the paroxysms of coughing assume an intermittent character, which is not unfrequently the case, the administration of an emetic at the moment when a paroxysm is expected, breaks the periodic habit, and facilitates the cure of the disease, on the same principle as in ague. I have, nevertheless, seen much mischief result from the frequent repetition of emetics in those of delicate and irritable habits; and when the stomach and the chest consent, if the expression be allowable, and the paroxysms are terminated by vomiting, the exhibition of emetics is not only unnecessary, but highly detrimental.

Practitioners are divided in opinion with respect to the kind of emetic which should be employed. Some imagine that by the employment of squills and other nauseating expectorants, as emetics, a double indication is effected; but as the operation of all expectorants is through the stomach, on which they must produce nausea before the air-tubes can be relieved by them, those emetic substances are the best which are most easily limited in their effects; and consequently ipecacuanha, combined with small doses of tartar emetic, is preferable to every other emetic.

The promotion of expectoration is undoubtedly useful in this stage of hooping cough; but, in very young subjects, it can prove beneficial only when accompanied with vomiting; and as emetics are not always advisable, other methods have been sought for, to promote this effect. Among these, I have seen the inhalation of both highly-diluted nitrous acid gas, and of the vapor of hot tar, tried with apparent benefit. The former was first tried by Mr. Patterson, surgeon to the Ferton Hospital, and is noticed in Dr. Carmichael Smyth's Report upon Nitrous Acid Gas; but the cases were too few to form a decided opinion of its powers; and in my own practice, I have seen it used once only: in that instance the patient rapidly improved. Mr. Patterson employed it under the supposition that it might neutralize the virus in the air-passages of the lungs, and thence render it inert, in the same manner in which it destroys contagious matter floating in the atmosphere: I used it with the hope of exciting the lungs to discharge the mucus, with which, in the case referred to, they were completely choked up. It certainly produced the desired effect; and after the employment of it for three successive days, the most striking improvement took place in the patient, who previously appeared to be sinking into his grave. I have had no other occasion for using the nitrous acid gas in hooping cough, but I feel much disposed to recommend it in similar cases. I have employed more frequently the inhalation of the vapor of tar, in less urgent cases, and can bear testimony to its effects in promoting the expulsion of the glairy mucus from the trachea. I may here mention that I have lately found it equally useful in a very distressing case of asthma. The simplest method of extracting tar vapor is to put the tar in a pipkin capable of holding triple the quantity intended to be used, and placing this in a vessel containing sand sufficiently heated to keep the tar in a state of gentle ebullition: it promotes expectoration, which is followed by sleep, from which the patient awakes greatly refreshed.

In this stage of hooping cough, when the excretions are not duly effected, it is necessary to resort to purgatives; but I must agree with Dr. Cullen in condemning their frequent employment.

In the second stage of hooping cough, which may be regarded as commenced when the febrile symptoms have abated, and the cough returns in regular paroxysms, such as in the cases now under treatment in this Dispensary, the symptoms are to be combatted by those medicines which, having sedative powers, allay the morbid irritability of the nervous system, and, consequently, act as antispasmodics.—This class of remedies is extremely extensive; and the greater number of the articles which it contains have been, at one period or another, employed in hooping cough; and although each has had its advocates for a certain time, yet many of them, either from the influence of fashion, which has a sway even over medical opinions, or from a conviction of their inutility, have fallen into disuse. The list is still sufficiently extensive; but I will notice those only of which I am able to speak from my own experience and observation.

The first of the tribe of narcotics, opium, has been extensively employed, but has generally been supposed to prove hurtful, by checking expectoration: this is true when opium is given alone, yet, in combination with the nitrate of potassa and ipecacuanha, or tartar emetic, I have found it answer every indication. In some habits, its effects upon the functions of the brain stand, certainly, in the way of its exhibition; but in these instances I have found the acetate of morphia prove beneficial; and were this remedy less expensive, it would, with the exception of prussic acid, render nugatory the employment of every other narcotic. I have given it, dissolved in the bitter almond emulsion, in doses of one-eighth of a grain, once in six or eight hours, to children five years old. I have not found the same beneficial results from the use of the lactucarium, or of hyosciamus, both of which have been highly extolled as sedatives in hooping cough.

Belladonna has been very extensively employed on the continent, as an antispasmodic, in hooping cough; and we might, *a priori*, augur well of its power from its general effects upon the nervous system. In Germany, the root of the plant has been greatly extolled. It has been given in doses of a quarter of a grain gradually increased to a grain, to children under one year of age; in doses of half a grain, at first, to children between the ages of two and four years; and in three quarters of a grain, to those above this period of life. I have had no experience in the use of the root; but my opportunities of witnessing the effects of belladonna have been numerous and satisfactory, and the cases now under treatment here will enable you to verify the opinions I have to offer you concerning it. I have generally given the medicine, at first, in doses of the tenth of a grain, to children of from two to five years of age; and, to insure the accuracy of the dose, have prescribed in the form of pills made up with crumbs of bread, which, as they are small, are readily swallowed in a teaspoonful of pap or gruel. In combination with the extract of belladonna, I have usually prescribed ten or twelve grains of the subcarbonate of potassa or of soda, and half a grain of powder of ipecacuanha, to be taken in an ounce of the bitter almond emulsion, between each dose of the pills. The result of this treatment has been the dimunition of the cough, both as to violence and the frequency of its recurrence; and except in a few instances, which I am inclined to refer to idiosyncracy, the plan does not appear to have been productive of the smallest inconvenience. When the dose has been too suddenly augmented, or when too large a dose has been given at first, it dilates the pupil, produces a paralysis of the retina, and a consequent temporary blindness, which continues two or three days after the medicine has been discontinued, but seldom occasions any other unpleasant effect. In one case, however, in which a boy, five years old, had taken the dose intended for his brother of nearly double the age, the head was singularly affected: a state of delirium, not unlike that attendant on mania, supervened; and although the attention could be easily roused, yet the mind immediately relapsed into its morbid state, and continued so for two days and nights, during which there was uninterrupted watchfulness. I generally continue the use of belladonna, gradually augmenting the dose, until a scarlet eruption covers the skin, when I stop until this disappears. Whilst this eruption is out, the cough ceases, and if it do not soon subside, the habit is so much overcome as to prevent the recurrence of it with as much severity as before.

Digitalis has been given as a sedative in this stage of hooping cough, but with various results. The unsuccessful instances, however, in my opinion, have arisen from the exhibition of the remedy under improper circumstances; nor do I think that the manner in which this powerful remedy operates on the animal economy is generally understood. Digitalis has been given to keep down the arterial action in hooping cough, as well as in mania,—an effect which I am satisfied it produces in neither disease, unless it be given in large and nauseating doses, and unless the arterial excitement be previously lowered. When this has been effected, when the pulse is feeble, and the habit in a state approaching to that of debility, or general relaxation, digitalis is a most certain and powerful sedative, calming nervous irritation, allaying spasmodic action, and inducing sleep. The striking effects of this article of the materia medica in mania, first induced me to employ it in hooping cough, and in delicate habits I have found it answer my expectations. The form of the remedy which I have usually employed is the tincture, which I give, according to circumstances, in small doses at first, gradually increased to an extent much beyond what is generally imagined can be safely prescribed. Thus, in hooping cough, I have carried the remedy to the extent of forty drops, three time a day, to a boy eight years of age; and in adults, laboring under mania, I have given it, with the best effects, in doses of 110 drops, once in eight hours, for ten days successively.

Alkalies and prussic acid act, nearly in the same manner, on the coat of the stomach, and through that organ of the general system. When taken into the stomach, their first effect is on the nerves of the viscus, the irritability of which they lessen in proportion to the extent of the dose, and communicate this effect by sympathy to the larynx. Prussic acid, in particular, is unquestionably the most powerful antispasmodic which can be employed in hooping cough; and it has the advantage of being equally useful in every stage of the disease,—the due apportionment of the dose and its combinations enabling it to answer the indications to be attended to in every stage of the complaint. Thus, in the first or febrile state, the prussic acid, given in medium doses of one or two minims to children of ten or twelve years of age, in combination with ipecacuanha and nitrate of potassa, moderates the fever; in the second or spasmodic stage, in larger doses, it allays the violence of the cough; and in the last stage in small doses of a single minim, combined with infusions of bark, it aids the tonic power of the bark by allaying the irritability of the stomach, and thereby enabling the gastric fluid to be secreted more slowly, consequently in a more natural state, and better fitted for the purposes of digestion. In one of the cases now under treatment, the dose is one minim only, owing to the great delicacy of frame of the child; but in the others, the dose is two minims, repeated every fourth hour.

Musk, conium, the sedum palustre, and acetate of lead, have been used by different physicians, and have been extolled; but I have had no opportunity of forming an opinion as to the efficacy of any of them, except conium, which has rather disappointed my expectations in hooping cough. I am well aware, however, that in the form of extract, much depends on the preparation of the remedy; and even in the form of powder, the manner in which the leaves are dried, and the powder is preserved, will greatly alter its influence as an antispasmodic.

Besides the employment of sedatives in this stage of complaint, stimulant remedies are also prescribed, with the view of subduing the propensity of spasmodic action in the trachea, by exciting, as Dr. Good remarks, "a general or remote local revulsion." But, unless as external applications, I have had no experience of the value of this class of remedies. The internal stimulants which have been employed in hooping cough are cantharides, ammonia, ether, camphor, myrrh, the herb paris, and the rhus *vernix*. The external stimulants are camphor, garlic, ammonia, oil of amber, turpentine, tartar emetic, and tincture of cantharides, all of which I have seen beneficial, when employed in combination with oil or soap liniment, in the form of embrocations; probably producing their good effects by stimulating the dorsal and cervical nerves, which are those chiefly concerned in the function of respiration.

That period, in the progress of hooping cough, in which the spasmodic state has somewhat abated, and the cough is kept up rather by habit than by the continuance of the operation of that virus which originally produced it, is regarded as its third stage. The frame of the body, and its powers exhausted by the previous diseased action, is morbidly susceptible of every impression which can keep up the spasmodic habit. In this stage, therefore, tonics are judiciously prescribed; and the preference is given to those which produce their effects most quickly, and are the least disagreeable to the palate.—Cinchona has been employed for this purpose since the days of Sir John Floyer, who lived in the seventeenth century; it is now superceded by the sulphate of quinia, which can be given to children in the form of pills, with as much facility as the extract of belladonna. I have employed the oxide and the sulphate of zinc, nitrate of silver, iron in various forms, and the arsenical solution, as tonics in this stage of hooping cough, and feel some difficulty in determining to which I should give the preference. Perhaps the best of all tonics, at this period of the disease, is change of air; and this, in conjunction with ass's milk and the mucilage of the lichen islandicus, I have seen restore children worn down to skeletons, and rapidly becoming tabial, when no other human means appeared likely to relieve the little sufferers.

The diet in hooping cough should be of a vegetable and farinaceous kind, until the second stage of the complaint be over; after which, light animal food may be allowed.

I have only further to notice that hooping cough is said to be cut short, in its progress, by vaccinations on the third week after the commencement of the hoop. This method of treatment was first suggested in Germany, and its efficacy is said to have been lately confirmed in America. Allowing that the observations of this influence of vaccination be correct, the remedy must always be of very limited utility, as it is not likely that vaccination should be delayed, with the risk of smallpox being taken in the interval, in order to keep it in reserve as a remedy in hooping cough.—*Boston Medical and Surgical Journal, May* 24, 1831.

19. *Case of Constitutional Disease, arising from a Simple Local Irritation.*—The following case, related by Dr. Rynd, in the 5*th Vol. of the Dublin Hospital Reports*, is extremely interesting, as *proving that a morbid poison may be generated in consequence of a local irritation, and that this poison may contaminate the entire system;* and therefore tends to overturn some of the unphilosophical notions even to this day entertained by some respecting the necessarily specific nature of a number of diseases. The subject of this case was a gentleman, aged thirty-eight, of a florid countenance, indicating a highly sanguineous temperament; rather corpulent, and not of very active habits. He had previously enjoyed good health, unless from occasional attacks of cynanche tonsillaris. He had been, during the past winter season, more than usually employed in anatomical pursuits, and about three weeks before he wounded himself with the pin, he received a slight cut on the thumb while examining a subject that had died of diseased hip-joint; the wound, however, had healed speedily and well. He has been married twelve years, has had nine healthy children, seven of which are living, *and by no accident or possibility could he have contracted any syphilitic taint.*

About three weeks after the first appearance of the fungus, the lymphatics of the forearm became inflamed; the inflammation extended along their course to the axilla; sixty leeches were applied, and the inflammation was subdued. About a month afterwards, an enlarged gland appeared in the axilla, which was also resolved by the application of leeches.

On the 27th of September the abscess burst, and on the 29th he went to the country, emaciated and debilitated to an extent not to be explained by the effects of so small a local disease. On the 10th of October his throat felt sore, with some trifling difficulty of swallowing; he returned to Dublin, his throat was examined, and no appearance of disease was discovered, except a very slight blush of inflammation, which soon passed away. Towards the latter end of October, an eruption of scarlet patches came out on his forehead, forearms, and slightly on the shoulders and inside of the thighs. Sarsaparilla and the nitro-muriatic acid were now prescribed, and taken in considerable quantity. In this state he remained until the end of December, his throat being on one day free from uneasiness, and perhaps on the next extremely sore, the eruption sometimes disappearing entirely, sometimes showing faintly under the cuticle like fading measles, and sometimes of a bright red scarlet colour. He continued taking the sarsaparilla up to this period, with warm baths twice a week, and his general health had been improving since the sore on the thumb had healed. He got weary of taking medicine, and now discontinued it, and trusted to a nutritive diet, and the use of port wine.

Early in January, 1829, an anomalous kind of eruption appeared on his scrotum, and produced great annoyance, it was so intolerably itchy; but it scaled off and disappeared in the course of a week, without the adoption of any medical treatment whatever. On the 15th, a tender tumefaction arising from periostitis was discovered on the left tibia; on the 19th it disappeared. On the 2d of February, an eruption similar to what had before appeared on the scrotum reappeared; it also went away without the use of medicine. During the months of March, April, and May, his throat could never be considered in a state of health, and exhibited so much variety of symptoms, both as to appearance and pain, as rendered the case inexplicable as it was curious; sometimes when there was no trace of inflammation visible, the pain would be excessive. An erythematous redness was, on the other hand, often suffused over the soft palate, and occasionally an appearance resembling abrasion of the surface, while there were no indications of exacerbation of pain. Sometimes whilst speaking, his voice would suddenly fail, and a paroxysm of cough interrupt his articulation; the cough was hard and unattended by expectoration; he would often commence eating, suffering under extreme torture in every attempt to swallow, and after a few moments he would perform deglutition without the slightest pain. In a word, these symptoms came and went alternately without any assistance being sought from medicine. But this uncertain and precarious state of health could not be borne any longer, and he determined to try the effects of mercury, though contrary to the advice of his friends, and took two grains of calomel daily for four days, when he was seized with a cough, accompanied with some hæmoptoe. The mercury was discontinued immediately, and never resumed: his mouth was made slightly sore by this quantity, his gums became spongy, and his breath foul. All the symptoms gradually and slowly declined, and he is now occasionally visited by the following:—Wandering pain in the hip joint of the right side, and weakness which prevents exercise on foot to any considerable extent. Some few pale and almost indistinct stains of eruption on his forehead after exercise. Some broad, flat patches of eruption of a scarlet colour, but not always of the same intensity, on the back of the hands: during the last winter the skin on the palms of his hands was hard and horny, and the natural lines appeared like fissures; this has now disappeared. His throat has never been sore since he took the calomel.—*American Journal of the Med. Sciences, August*, 1831.

20. *Poisonous Effect of the Blood of a Patient upon Leeches.*—Dr. Graves, in a clinical lecture delivered at the Meath Hospital, Dublin, and which is published in the *London Medical Gazette* for February last, related the following curious case. A woman was admitted into the hospital for an obstinate stomach affection, which had many of the characters of organic disease, but the result proved that no permanent alteration of structure existed in the stomach, for she completely recovered. This patient at one period of her complaint was afflicted with constant nausea and vomiting, accompanied by considerable tenderness in the epigastrium, for the relief of which twelve leeches were applied to the region of the stomach. Shortly after they had begun to act, they fell off, and immediately died. This curious circumstance naturally arrested attention, and twelve leeches more were ordered, the precaution being first taken of perfectly washing the skin to which they were to be applied. These leeches shared the same fate as their predecessors, and this happened in several successive trials, made by way of experiment, until about sixty leeches, all previously in good health, had been thus destroyed. Of course there is no other way of accounting for this phenomenon, than by supposing this woman's blood was so different in composition from human blood in general, that it proved poisonous to these animals. No accurate chemical experiments were made to ascertain what was the nature of this poisonous principle, but it may be observed that the physical properties of her blood presented nothing remarkable, its serum and crassamentum having the ordinary colour, consistence, and smell.—Neither could this quality have been owing to any medicine she was at the time using, for the blood continued to act as a poison to the leeches for several days after she had altogether left off medicine. It was suggested by some of the students, that the poisonous property might have arisen from a small quantity of hydrocyanic acid which she had been taking, but the remark just made disproves the truth of this hypothesis. Besides, we have frequently had patients in the hospital in whom much larger doses of hydrocyanic acid were not followed by this state of the blood. In reference to this it may be also observed, that the lower orders of animals seem much less susceptible of the effects of certain poisons which act upon the nervous system, than the higher orders.—*Ib.*

21. *Case of Spontaneous Lactation at an advanced age.*—The following example of this curious and interesting physiological phenomenon, is related in a recent number of the *North of England Medical and Surgical Journal*, by George Semple, Esq., of Shipley Hall. Mrs. B., æt. 49, mother of 8 or 9 children, the youngest of whom is about 12 years old, lost, a year ago, a daughter-in-law, who died of peurperal inflammation about a fortnight after confinement of her first child.

On *her* death Mrs. B. took the charge of the infant—a little, puny, sickly baby. The child was so fretful and uneasy, so averse to taking any kind of food, and so troublesome, that Mrs. B. after several sleepless nights was induced, by way of soothing, to permit her to take the nipple of her breast into the mouth—the child was pleased and soon sunk to rest, and the old lady of course continued to give her this cheap and innocent sedative, from time to time. In the course of from thirty to thirty-six hours she felt very unwell, her breasts became extremely painful, considerably increased in size, and soon after, to her utter astonishment, the lacteal fluid was secreted, and poured forth in the same abundance as on former occasions after confinement of her own children.

The child, now a year old, is a fine, healthy, thriving girl, and only a few days ago, says Mrs. S., I saw her eagerly eugaged in obtaining an apparently abundant supply of healthy nourishment from the same fountain, which nearly twenty years since poured forth its resources for the support of her father.

Mrs. B. is a stout, healthy woman, and has continued to menstruate regularly, both since weaning her last child, nearly eleven years ago, and during the time she has suckled this little grandchild.

22. *Congenital absence of the Gall-bladder.*—M. Amussat communicated to the Royal Academy of Medicine, the case of a man, twenty-four years of age, who died from a white swelling of the knee, and in whom, on examination after death the gall-bladder was found entirely wanting: the ductus choledocus was formed by two large canals.

The total absence of the gall-bladder is an extremely rare occurrence, and one the possibility of which has been doubted by several anatomists: it has been, however, met with.—*Gazette Medicale, 26th March*, 1831.

23. *Two newly-discovered Muscles for compressing the Dorsal Vein of the Penis, in Man and other Animals.*—Dr. Houston has described, in the fifth volume of the *Dublin Hospital Reports*, two muscles situated between the arch of the pubis and the penis; and for which he proposes the name of *compressores venæ dorsalis penis.*—These muscles are more distinct in most of the mammalia, than in man. In the latter, they arise from the rami of the pubis, above the origin of the erectores penis and crura, and ascending in a direction forwards, are inserted above the vena dorsalis by joining with each other in the median line. They form a thin stratum of muscular and tendinous fibres, about one inch long, and three-quarters of an inch broad, and may, perhaps, be looked upon as portions of the erectores penis, which, instead of being inserted into the sides and lower part of the corpora cavernosa, mount over those bodies, to exert their compressing influence on the vena dorsalis. They enclose between them and the penis, the vein, arteries, and nerves of this region. Their anterior fibres are distinguished from those of the erectores, by the fibrous attachment of the crura to the pubis; their posterior margins are kept distinct from the front part of the levatores ani, known under the name of Wilson's muscles, by the pudic artery, which divides them in its course towards the dorsum of the penis.

The best procedure to display these muscles is the following:—Detach the bladder and levator ani, with the hand, from one side of the pelvis; then divide with a saw the pubis and ischium about one inch from the symphysis, and break off the bones at the sacro-iliac articulation: next dissect away carefully the remaining portion of the pubis from the symphysis, periosteum, and crura penis, and then the compressores venæ, bearing still their natural relation to the crura and other muscles, may be exposed with very little difficulty.

The insertion of the muscles being in a great measure outside the pelvis, they may also be demonstrated without the section of the bones, by cutting on them in front of the pubis, and looking carefully for their tendon at the side of the venæ dorsalis: from the tendon the knife may be carried downwards and backwards in the course of the fibres, and nearly the whole of the muscles can be thereby exposed.

It must however be remembered, that it will be needless to search for them in a thin emaciated individual, where the other muscles of the perinæum are so pale and soft, that even they can scarcly be distinguished. The subject should be robust, and the muscles red, in order to demonstrate them.

The use of these muscles is evidently to compress the dorsal vein of the penis, and mechanically obstruct the return of blood.

24. *Taliacotian Operation for Restoration of the Under Lip.*—By C. Bryce, M. D.—In July last, the Hakim-Bashi sent me, at Constantinople, the following singular case of destruction, by mortification, of the under lip and chin, which, from the success following the treatment pursued, seems worthy of notice.

The subject of it, a Turkish child, three years old, was attacked, in June preceding, with scarlet fever, that spread sparingly over the body, and in which the mouth and throat were aphthous, but not ulcerated, disappearing by the use of a wine gargle and laxatives. The subsequent affection of the mouth was preceded by much fever and restlessness, and appeared in a small pustule on the left commissure of the mouth, which became rapidly black, and open, discharging an offensive sanies; the gums at the same time were discolored, and bled on the child's crying. The sore formed on the outside of the mouth spread quickly downward and laterally; and on the seventh day of the disease, when the patient was first brought to me, the whole of the under lip and chin presented a fetid, black, irregular-shaped slough, surrounded by a broad, dark-colored line of inflammation, marking the threatened progress of the disease on the integuments, the left cheek, and upper lip, which were painful and hard. The incisors of the lower jaw were loose, their gums and alveoli partaking of the affection, and by contact, involving the lip and anterior edges of the tongue. The tongue was œdematous and loaded, but the fauces showed no appearance of unhealthiness. The countenance had a peculiar livid color; body dry and hot; pulse small and rapid; diarrhœa.

From certain circumstances, an emollient poultice to the sore, and frequent ablutions of the mouth, and a few drops of laudanum internally, were all that could be recommended at this period. On the third day's visit, the tenth of the disease, observing the rapid progressive destruction of the integuments, the gangrenous parts were touched with diluted nitric acid, and sulphate of quinia with opium, were exhibited. This external treatment, twice renewed in thirty-six hours, did not in any measure arrest the spreading of the mortification, which had now involved an inch and a half in circumference of the left cheek and upper lip, and extended downwards under the chin. The diarrhœa was checked, and the child had enjoyed some hours of sleep, but was evidently sinking. It was now determined on to apply a fermenting poultice over the diseased parts, and, and to administer pills composed of quinine, camphor, and hyoscyamus, in large doses, and allow 8 oz. porter daily. This plan, after forty-eight hours' continuance, showed a marked improvement in the patient: the mortification had made no further progress for the last eighteen hours; the slough was partially detached; whilst the look of the child was improved, with a slower and a fuller pulse. The poultice and porter were continued as before, as were the pills, reduced in strength. In a few days the slough was totally separated, leaving a florid granulating surface. The integuments had assumed their natural color and feel; four incisors and two canine teeth, with fragments of alveoli, were removed, as were also three small exfoliations from the under maxillary bone. The tongue had thrown off its slough, and appeared healthy.

The only treatment practicable for the moment, was to encourage granulations by spirituous applications, and support and diminish the loss of substance by compresses and bandages, whilst the general health of the child was improved by liberal diet and change of air. In six weeks a healthy cycatrix had formed, and the breach of continuity was much lessened by its contractions. It was now very evident that a complete, or even partial, reparation of the great destruction of the soft parts, could be promised only by an operation, the necessity for which arose as much from the disagreeable deformity of the face, as from the evident ill effects on the system that the constant flow of saliva occasioned. The writer was encouraged to this attempt by the very perfect success of a Taliacotian nose, made a short time before by him at Constantinople. The operation, in which he was assisted by the advice and skill of his intelligent friend Mr. Millingen, was performed by removing from the upper part of the throat a triangular portion of the integuments, whose suitable form and size had been judged of, by measuring a model of the lip and chin adapted to the deficiency. This segment was now reflected, the twist being made immediately on the point of the chin, and its two angular points attached by ligatures to the commissures of the mouth, previously made bare by scarifications, as was also the whole of the former cicatrized surface. The flap was further supported by adhesive plaster and bandage, and the wound on the throat brought together by the same means. The parents were directed to keep the child in a recumbent posture, and to feed her sparingly on pap. The wound was looked at on the third day;—on the fifth the dressings were changed, when adhesion had taken place on one side very completely, and on the other, although the ligatures had cut through, there was no opening of the wound: there existed no symptoms of excessive inflammation of the parts, or of irritation of the system. In fifteen days the cicatrices were perfectly formed, and the wound on the throat almost closed. On closing the mouth, the artificial lip seemed very well adapted to the other, and even when partially opened retained the saliva, and very materially diminished the unseemliness of features. It cannot be yet determined whether or not the second set of teeth, with their alveoli, have been so much destroyed as to prevent the great help derivable from their growth, by supporting the flaccid flap. On the whole, the result of the operation was very satisfactory, especially at Constantinople, by exalting the practice and utility of surgery amongst the Turks, and encouraging them to submit to operations beyond the barber's province of bleeding and tooth-drawing.—*Glasgow Medical Journal.*

25. *Pathology of Jaundice.*—The appearance of the skin and the nature of the discharges in jaundice, have given rise to the idea that an obstruction takes place in the ducts, by which the bile is prevented from passing into the duodenum. Cases of the disease are so seldom fatal, as to afford but limited opportunities of settling this point by actual examination. Seven cases, however, terminated fatally in the wards of La Charite in Paris, in 1828, 1829, and 1830, in which jaundice existed in a severe form. On examination, six of the seven were found to present no obstruction whatever of the biliary ducts. In the seventh they were impervious, being involved with the gall-bladder, part of the liver, and part of the pancreas, in a cancerous mass of the size of the fist. In several of the cases, the liver is said to have appeared of a pale yellow color, and in one only to have contained an unusual quantity of bile.—*Bost. Med. and Surg. Journ.*

26. *Living Skeleton.*—Whilst that miserable being, by the name of Edson, who was known here under the above title about a year since, was exhibiting himself in Paris, it appears that with the view of extending his reputation, he offered himself as an object of curiosity to the Academy, who appointed M. Larrey to examine him, and report accordingly. The distinguished academician not being able to attend to this duty, his confrere, M. Arogo, undertook it in his stead. His report was read at the meeting of the Academy, the 21st of December last. As his condition in one or two respects seems to have altered since he left here, it may perhaps be interesting to those of our readers who recollect his person, to see an abstract of M. Arago's remarks. After speaking of the general facts regarding his place of birth, early history, &c., the reporter arranges the most remarkable circumstances of his condition in the following manner:

1. There is a reduction of the bulk of the person in all its dimensions, even the height being less than when in a healthy state.

2. The muscles, which can be perfectly well examined through the skin, appear as small, dense cords, being deprived, to a great degree, of their interstitial cellular substance.

3. The heart, which beats very feebly, is smaller than a natural size, and seems to have partaken of the general emaciation.

4. The teeth are rendered naked by the absorption of the gums, and some of the incisors are wanting. He has been affected with chronic ophthalmia, which has terminated in ulceration of the cornea, so that he is now entirely blind.

5. The excretions are regular, but small, and in proportion to the quantity of food taken. The urine is turbid.

6. The hair is thin and nearly white.

7. The senses, with the exception of the loss of sight above mentioned, remain unaffected. The mind is clear, and he gives proof of considerable capacity.

8. The reproductive powers are not impaired. He has four children, all in excellent health.—*Ib.*

27. *Poisonous Secretion from the Body of a Horse.*—A horse in the town of Benton, in this county, was discovered to be ailing by his owner, and considerably bloated. Incisions were made through the skin in different parts of the body, from which proceeded a kind of liquid matter resembling honey. The appearance was not so singular as its poisonous qualities. We learn that those attending him, not apprehending danger, were less cautious about diffusing it upon the hands and arms. Wherever it touched, it produced a more surprising effect. The skin first blistered, and then seemed to yield to the poison by breaking away, and presented the flesh in a cankered state. They were alarmed, and immediately called medical aid, which, we are happy to learn, was so successful as to stop its ravages. The horse still lives.—*Penn. Yan. Gaz.*

28. *The Use of Sulphate of Morphine in Ophthalmia.*—By CHARLES A. LEE, M. D., *New-York.*—I have lately employed a solution of the sulphate of morphine, in cases of ophthalmia, with very beneficial results. The first case in which I used it was one of acute conjunctival inflammation, attended with great intolerance of light, with constant pain and itching. The relief was immediate, and no other wash was required. Every practitioner who has had much experience in this class of diseases, knows the efficacy of the lotions in general use. For the most part, by causing irritation, they do more harm than good. Even the aqueous solutions of opium, the most sedative of any in use, are liable to this objection, as is remarked by Francis, in his Synopsis.

The advantages which morphine possesses over all others, recommended for eye lotions, are too obvious to need pointing out. As a direct sedative, it allays all pain and irritation, and diminishes that distressing intolerance of light, which usually attends ophthalmic cases. My usual formula is the following:—R. Sulph Morphine, gr. ij.; Aquæ dist. dr. i. M.

Some cases require a solution of greater strength, according to the severity of the symptoms. It should be applied tepid, and occasionally a cloth, dipped in the solution, may be kept applied to the eye. I cannot state, from my own experience, whether it will prove as useful in chronic as acute cases, though, doubtless, it may be used advantageously to answer the same indications. From the atony of the vessels in these cases, stimulating lotions of the metallic salts will be indispensable.—*New York Med. Journal.*

29. *Veratria and Colchicum Autumnale.*—The next alkali with which Dr. Bardsley has furnished us the result of his experience, is the veratria, and the preparation used by him was the acetate. The reader will recollect that the substance known by the name of veratria, was first obtained from the veratrum *sabadilla* by those French chemists whose names were mentioned as having discovered the strychnia and brucia in nux vomica. The same indefatigable analysts afterwards discovered the substance under consideration, in the veratrum *album* and in colchicum autumnale; and the evidence of its activity was exhibited in its rapidly fatal effects on dogs and other quadrupeds. As a medical agent, Dr. B. first tried the acetate of veratria in the following case of *dropsy.*

Hugh Cairns, 39 years of age, was admitted into the hospital on the 7th of November, 1826, on account of ascites and anasarcous swellings of the lower extremities. He had been ill three months.—His urine was scanty, thirst urgent, pulse feeble, and respiration somewhat difficult. He commenced with a quarter of a grain of veratria every four hours. This quantity did not act upon the bowels with energy. It was then increased to have a grain in the same intervals. In this proportion it occasioned several copious watery dejections daily. On the 30th, the swellings were considerably diminished. He was now directed to take a grain of veratria twice daily. On the 9th of January he was discharged, cured.

The hopes encouraged by the termination of this case, were subsequently disappointed by the total failure of the remedy to do good in any other of the 6 cases of dropsy in which it was administered, and in two of which relief was afterwards obtained from the calomel and squill. It appears, therefore, so far, to be of little value in medicine, and in no respect advantageous over elaterium, with which it appears to correspond very accurately in its effects on the human system.

The most valuable part of Dr. B.'s experiments on this head, are his trial of veratria, and of colchicum, each in 24 cases of *chronic rheumatism.* The fame of the meadow saffron in the cure of gout, rheumatism, and some pectoral complaints is so high and extended, that the degree in which its efficacy may be attributed to this active principle which has been discovered in it, is matter of interest to every practitioner. The cases of rheumatism, to which Dr. B.'s observations with the veratria appear to have been confined, were very similar to those in which he administered the colchicum;—so much so as to be regarded by him parallel.

It is well known that hellebore and meadow saffron have each been suffered to be the basis of the famous eau-medicinale, which was long esteemed a specific for gout, and which exerted a power equally curative over its near relative, the rheumatism. The prescription from which this French nostrum was compounded, became extinct with its author—a fact which is never referred to by the bon vivant but with a solemn countenance, and which may perhaps be regarded by all as matter of regret. It will appear by the following table, that it will be hard to decide, from their effects on the system, which of the two simples above mentioned most probably gave its efficacy to the remedy of M. Haller. The form of colchicum used by Dr. B. was the vinous tincture prepared from the seeds.

24 CASES OF CHRONIC RHEUMATISM TREATED

1*st, with Veratria,* 2*d, with Colchicum.*

| Species. | Treatment. | Result. | Species. | Treatment. | Result. |
|---|---|---|---|---|---|
| Sciatica | Veratr. gr. 1-4 ad gr. ss. ter in die | Relieved | Sciatica | Vin. Colch. gutt. xxv ter in die | Relieved |
| Chronic Rheumatism | Ditto | Relieved | Chronic Rheumatism | Ditto | Cured |
| Sciatica | Ditto | Cured | Ditto | Ditto | Relieved |
| Lumbago | Ditto | Cured | Lumbago | Vin. Colch. gutt. xv ter in die | Cured |
| Ditto | Ditto | Cured | Ditto | Vin. Colch. gutt. xxv and xxx ter in die | Cured |
| Chronic Rheumatism | Ditto | Relieved | Chronic Rheumatism | Vin. Colch. gutt. xxv ter in die | Cured |
| Ditto | Ditto | Cured | Ditto | Ditto | Relieved |
| Ditto | Veratr. gr. 1-4 ad gr. i., bis in die. | Relieved | Ditto | Ditto | Cured |
| Ditto | Veratr. gr. 1-4 ad gr. ss. quat. in die | No benefit | Ditto | Ditto | Relieved |
| Ditto | Ditto | Relieved | Ditto | Vin. Colch. gutt. xv ter in die | No benefit |
| Ditto | Ditto | No benefit | Ditto | Ditto | Ditto |
| Ditto | Veratr. gr. 1-4 ad gr. ss. ter in die | Ditto | Ditto | Ditto | Ditto |
| Ditto | Ditto | Ditto | Ditto | Vin. Colch. gutt. xx ter in die | Relieved |
| Ditto | Veratr. gr. 1-4 ad gr. ss. quat. in die | Ditto | Ditto | Vin. Colch. gutt. xxv ter in die | Relieved |
| Ditto | Ditto | Relieved | Ditto | Ditto | Ditto |
| Sciatica | Ditto | Cured | Ditto | Ditto | Cured |
| Chronic Rheumatism | Veratr. gr. 1-4 ad gr ss. ter in die | Relieved | Ditto | Ditto | Relieved |
| Ditto | Ditto | Ditto | Sciatica | Ditto | Ditto |
| Ditto | Veratr. gr. 1-8 ad gr. ss. bis in die | No benefit | Ditto | Ditto | Ditto |
| Ditto | Veratr. gr. 1-4 ad gr. ss. ter in die | Relieved | Chronic Rheumatism | Ditto | No benefit |
| Ditto | Veratr. gr. 1-4 ad gr. ss. quat. in die | Cured | Ditto | Ditto | Ditto |
| Ditto | Ditto | Relieved | Ditto | Ditto | Ditto |
| Ditto | Veratr. gr. 1-4 ad gr. ss. ter in die | Cured | Ditto | Vin. Colch. gutt. xxv ad xxx ter in die | Relieved |
| Ditto | Ditto | No benefit | Ditto | Ditto | Cured |

By comparing these tables, it will be seen that the Veratria cured 7—relieved 10—and failed in 7;—the Colchicum cured 7—relieved 11—and failed in 6. The coincidence is very remarkable, and leaves, in favor of the former, the advantage only of the convenience of administration from the small bulk in which it is given;—but the result of these cases appears to show that the efficacy of the meadow saffron is chiefly by virtue of the veratria contained in it.

The action of the two substances on the system was also analogous, both producing copious watery evacuations; and it is probable the claims of the saffron to a specific for gout, rest, in fact, on its power of producing such evacuations.

In the administration of both these medicines, Dr. B. noticed the pulse in a short time to become slower and depressed, and if the dose was rapidly augmented, distressing nausea and vomiting as well as purging. Yet he discourages its use in acute rheumatism, and considers the practice of Mr. Haden, who reports so favorably of it in this disease, as an exceedingly dangerous precedent. It will not perhaps be out of place here, to allude to the great and decided power of prussic acid in arresting vomiting, which is thus often the effect of large or continued doses of the colchicum. One or two minims of the acid, repeated two or three times a day, will seldom fail to arrest vomiting, however long it may have resisted other remedies. This fact must now be considered so well established as to require no further confirmation.

The dose of veratria is one-fourth of a grain, gradually increased to a half or whole grain twice or thrice daily. Of the wine of the seeds of the colchicum, the commencing dose in fifteen minims, and it may be slowly increased to thirty;—beyond these maxima, the stomach will rarely tolerate these powerful agents.

30. *The late Mr. Abernethy.*—The following brief account of this eminent surgeon, accompanies the notice of his death in the last No. of the London Medical Gazette

Mr. Abernethy died at his seat at Enfield, on Wednesday, the 20th, between the hours of four and five in the afternoon. The event, we must confess, took us rather by surprise; though from the circumstance of his having retired some time since from business and the bustle of the town, on the plea of bodily infirmity and declining health, we should not have been quite so ill prepared for the intelligence.

Thus has one of the brightest stars of British surgery vanished from our horizon: we have lost the lineal successor—the immediate link that connected us with the times of Pott and John Hunter—the Haller of England—one of the ablest physiologists of our day.

We may be pardoned for applying to him the pathetic lines which were originally appropriated to one of his predecessors:

"Cold is that hand which nature's paths display'd;
Dead are those lips on which instruction hung,
Fix'd are those eyes, enlivening all he said,
Forever mute is that persuasive tongue!"

It was the boast of Mr. Pott, the year before he died, that he had served, man and boy, in St. Bartholomew's Hospital for half a century. Mr. Abernethy could lay claim to the same distinction: he was literally worn out in the service. He had not attained to the "days of the years of man's life," neither had he reached the ordinary average, which, it is remarkable, is generally allotted to eminent men in our profession—to anatomists more especially: he was only in his sixty-seventh year when he sunk under the disease—an asthmatic affection (complicated, we suspect, with disease of the heart*), which for several years, but particularly for the last two, has been bringing him down gradually to the grave. Nothing, we believe, worthy of any special

* The body, we understand, has not been opened.

notice occurred during the latter stages of his complaint - it was attended with general dropsy, and proceeded steadily to the extinction of the vital powers. One circumstance, however, connected with the severe attack which he suffered two years ago—that paroxysm which broke him up, and forced him to part forever from his beloved chair—may be here related.

We have many illustrations on record of original and powerful minds displaying the influence of their ruling passion when almost in the mortal struggle. Haller is said to have felt his own pulse, and marked some phenomena, attending the last beats of fluttering existence; and John Hunter, amid the anguish of angina pectoris, scrutinized his symptoms with the eye of a pathologist. With Abernethy the lecture-room is well known to have been the scene of his chief delight; and in an attack which threatened him with suffocation, after some hours of struggling for his life, it is said the first words he uttered were, "egad, I think I shall still be able to lecture this afternoon!"

Full half a century has elapsed* since the walls of St. Bartholomew's first received John Abernethy as a student. The progress which he made in his studies was exemplary: he had scarcely attained his twenty-second year when he became assistant-surgeon to the hospital, and succeeded Mr. Pott as lecturer in anatomy and surgery.

Nor was this any trivial period for earning a character; it was no vulgar time for becoming distinguished. William Hunter had just gone off the scene, after having gained immortal reputation both as a teacher and practitioner; and John was still in the full vigor of his powers and his fame. Yet was not the young Abernethy undistinguished: already had he given birth to anticipations the most sanguine. He could not have been above thirty, when we find Mr. Earle (afterwards Sir James) speaking of him as one "who, whether he was considered as a practioner of surgery, or a philosopher, deserved to be mentioned in the most encomiastic terms;" and the very year after (1797) we find him, along with Sir James himself, honored with a niche in the temple of fame:

"——Call Earle useful, Abernethy deep"—

says the author of the Pursuits of Literature—the learned unknown—who thus, too, qualifies him in a note:—"John Abernethy, Esq., F. R. S., a young surgeon, of an accurate and philosophical spirit of investigation, from whose genius and labors I am led to think the medical art and natural philosophy may hereafter receive very great accessions."

Thus early did he give promise of that fulness and maturity of merit, which he afterwards so conspicuously displayed.

He began to teach his profession, it may be observed, at a time when others are usually employed in learning it. But this was not all; he soon after presented the world with some of those essays on

---

*Mr. Abernethy, we are informed, was sprung from Irish parents, but born in London in the year 1765. He was grandson of the celebrated presbyterian preacher, the author of the "Sermons on the Attributes."

various subjects connected with his profession, which placed him in the very first rank of physiologists, as a man gifted with superior powers of observation, and with extraordinary clearness and originality of thought.

It was in one of those essays that he first announced his plan of putting a ligature on the external iliac artery; thus showing the practicability of effecting what was hitherto deemed impossible; and thus making good his title to be esteemed as a benefactor and preserver of his species, by saving the lives of numbers who must but for him have inevitably perished. The operation is classed by M. Roux, along with that of John Hunter, for popliteal aneurism, and both set down as a brilliant epoch in the history of the progress of surgery. It procured Mr. Abernethy, in fact, a European reputation. Yet it may with much propriety be doubted whether the circumstances attendant upon this case entitle the subject of our present article to the honors of a philosophical inventer. The great operations of tying the external iliac, and of securing the carotid artery—which latter also he was the first, at least in this country, to perform—were the offspring of emergencies. They were, however, the applications to practice of preconceived theoretical knowledge; and it was this turn in Mr. Abernethy's mind which made him so constantly refer to Hunter. The peculiar character, in fact, of his professional pursuits, was the application of physiology to the improvement of practical surgery.

In process of time, he succeeded Sir James Earle as physician to St. Bartholomew's; and it is universally known to how high a pitch of public estimation that establishment arrived, both as a hospital and a medical school, while his name was connected with it. We cannot enter into any very minute particulars in this rapid sketch, but we may allude to one circumstance, relating to St. Bartholomew's Hospital, at the period in question—the surprising paucity of operations in that establishment during Mr. Abernethy's time. We say, emphatically, during Mr. Abernethy's time; for to him has the circumstance been by common consent attributed. Of the fact itself there can be no doubt; it has been frequently announced in the most public manner, by one of the highest authorities at present connected with the hospital, that there are not nearly so many operations now performed in St. Bartholomew's as there were five-and-twenty years ago.

In this respect Mr. Abernethy will strongly remind us of Haller and John Hunter—the gods of his own idolatry; and, perhaps, were we appointed to assign him a suitable rank among the eminent of his profession, we should place him in somewhat of an intermediate position between those two greatest physiologists that the world has ever seen. Haller, indeed, was a physician, but he was probably the most surgical of physicians; while Abernethy was, perhaps, the most medical of surgeons. And John Hunter, we should say, was more of the pure surgeon than either. All were sensitively cautious, averse to the performance of operations, except in the very last resort; and in all, it may be attributed, perhaps, to their possessing, in an eminent degree, those "compunctious visitings of nature" which are so beautifully becoming to the great and benevolent mind.

Haller had almost a monastic abhorrence of blood. John Hunter held that a surgeon "should never approach his victim but with humiliation:" an operation, he considered, as a "reflection upon the healing art; the habitual operator, as a savage in arms, who performs by violence what a civilized person would accomplish by stratagem." Mr. Abernethy is known to have entertained much the same sentiments: he specially praises John Hunter for being averse to operations, and imputes it to him as one of the excellencies of his nature. Nor was his own averseness a factitious feature in his character. We remember on one occasion, not many years ago, when Mr. Abernethy had to operate on a patient for stone, the requisite incisions were made by him with all due skill and adroitness; but the business was far from being completed when his stock of *sang froid* manifestly failed him: he tried in vain to grasp the stone; he could not find it; he became embarrassed; he hesitated; and at last anxiously transferred the forceps into the hands of his assistant, plainly showing, at least, how little of the studied apathy of the stoic there was about him. He never operated for the stone again.

And here we cannot help reflecting how much for the worse opinions seem to have changed of late—indeed very lately—with regard to the duties of a practical surgeon. Operations are beginning once more to be looked to as the test of pre-eminent ability: and a surgeon, if he go not through his work with something like what Gibbon calls "the savage coolness of an anatomist"—with more regard to scientific and dexterous performance, even than to the life of his patient—is but too frequently supposed to be possessed of only an inferior degree of merit. But we have just cited three of the greatest authorities of latter times: and it should never be overlooked that the proudest boast of modern surgery is, that of diminishing the number of its operations.

Mr. Abernethy derived much celebrity from his lectures: he was certainly, in his day, the most famous teacher in London. And it could scarcely be otherwise. He began early, and in a good school; he was a fluent and energetic speaker, and having a clear and accurate conception of what he taught, he knew well how to place in a distinct and intelligible point of view even the most abstruse subjects of discussion. The spirit which he threw into his delivery, together with his felicitous and striking powers of illustration, gave also a peculiar charm to his manner.

But in what terms should we notice that native and rich humor which not only carried him to the bosoms of his pupils, but rendered him so marked a favorite with the public? Perhaps since the days of Dr. Radcliffe there has not been a medical practitioner so popular, and so much run after, both for his acknowledged skill and on account of his eccentric peculiarities. But how contradictory are the reports which are current on this subject! Many of his patients, no doubt, have come away from him affronted, and with their self-importance seriously disturbed; yet how many have still been found anxious once more to try the fate of their reception! What were these, however, to the numbers who visited him and took their leave impressed ever after with the strongest sense of his humanity, his superior talent, and his perfect good humor? Liberality, in fact, and kindness, were among the most prominent features of his character; and his humor, though sometimes remarkable for its causticity, was never disfigured by that coarseness which belongs to the innumerable anecdotes falsely attributed to him, and many of which are more remarkable for their brutality than their wit. We could readily swell our little narrative into a book full of his facetiæ and humorous ebullitions, but that the solemnity of the present occasion forbids even an approach to the levity of amusement.

As we have just been speaking of his success with the public as a practitioner, it is but right to add, that those who have never seen Mr. Abernethy till of late years, can scarcely form any adequate idea of him in the brighter period of his life. Had this great man condescended to adopt the arts too often practiced for the purpose of getting money, what wealth might he not have accumulated! And having alluded to the accumulation of wealth, we are naturally led to another reflection on a subject somewhat metaphorically akin to this. Had the industry of Abernethy but equalled his genius, what treasures in science might he not have amassed! We have sometimes heard it laid to his charge, that his lectures in anatomy were rather too superficial—that he trusted, on many occasions, rather to the resources of his memory or his imagination, than to impressions derived from recent study and research—and that, as an author, Mr. Abernethy certainly might have done more. Both these imputations, however, are perhaps more visionary than real. If there were any semblance of truth about the first, the very imperfection imputed will be found to be not without a reasonable defence. The omissions in the matter of anatomical minutiæ were in many instances made advisedly; the lecturer probably thinking that such information was most properly to be acquired in the practical business of the dissecting-room, while the time thus saved would be most profitably employed in building up a sound surgical superstructure. And in laying down principles, what teacher ever surpassed the subject of our memoir? Then as to the deficiencies of his authorship, it is true that what he has published is not voluminous, but it is the concentrated essence of what might have been voluminous—the pith of the profound and extensive meditation of his earlier life. For our parts, we suspect had Mr. Abernethy had more time, and we are willing to add, more industry—consistently with his expressed principles—he might, in subsequent editions, have materially reduced the volume of his works. The motto, it may be mentioned, which he has adopted for one of his latest publications, is strongly indicative of his feeling on the subject:—"The increase of knowledge," says he, "is not like that of other things, for it is often accompanied by a considerable diminution of its bulk."

One anecdote, and we have done. Mr. Abernethy's memory was wonderful: no one knew when or how he formed his acquaintance with Shakspeare, yet he had the writings of the immortal bard almost by heart, and used very frequently to quote them. He was fond of the theatre, too, and would himself sometimes try his skill in the histrionic art: on one of these occasions, when in company with Mrs. Abington, he burst forth in the language of Richard, and the celebrated actress whom we have just mentioned declared that she had never heard any one whose manner and delivery so much reminded her of Garrick.

Much of the intellect and humor which belonged to him were displayed in his countenance. The best likeness of him is that painted by Lawrence, from which an excellent engraving has been made;—it represents him in his favorite occupation of lecturing. Another, by Turner, which shows him at a later period, is possessed of merit; it has been copied in mezzotinto. There is also a small print which exhibits him standing with his hands in his pockets:—the general expression in this design is very happy, though the individual portions of the picture are less felicitous.

To the Editor of the Boston Medical and Surgical Journal.

31. *Case in which brass wire was extracted from under the skin of a female, in whom it had induced Tetanic Symptoms.*—Dear Sir:—As the following case, from the singularity of the circumstances that attended it, may be deemed worthy an insertion in your Journal, I take the liberty of sending it you for that purpose.—Yours, &c.

JOHN B. BROWN.

Mrs. A., a young married woman, about 25 years old, of feeble constitution, and lax, moveable fibre, sent for me in the night of the 31st of January, 1831.

I found her sitting in bed, very much bent forward, and unable to alter her position, so much even as to look up. The account she gave me, was, that she had been subject to cramp in her lower extremities; and that then she had it all over her, in consequence, as she supposed, of a sudden cold, taken by walking on snow and ice with thin shoes. There was nothing impossible or very improbable in this statement; and as I was not made acquainted with any other more rational way of accounting for Mrs. A.'s present symptoms, I looked upon it as an unusual and singular case. I prescribed an emetic of tart. antimonii, which I supposed would relax the spasms, as it happily did, as soon as the medicine began to nauseate the stomach; and in the course of half an hour, she was able to extend herself and lay in a horizontal position as usual.

Feb. 1st, 9 o'clock, A. M.—Found Mrs. A. very comfortable. She had had a good night and slept well. Her pulse was a little accelerated: but her skin was moist, and there had been no recurrence of cramp. Gave a cathartic.

Feb. 2d.—Found Mrs. A. as well as yesterday. The medicine had operated favorably. As I was about taking my leave, she rather incidentally drew my attention to a circumstance, which she did not herself appear to consider of much importance, but which led me to suspect that the spasmodic symptoms she exhibited, at my first visit, were of a tetanic character. She proceeded to state, that she had something in her side, which she could distinctly feel, and which was moveable. She supposed it to be a piece of bone, splintered from one of the ribs, by a fall that she had on the ice, about two years previous—that she felt it soon after this accident, and that it had at times given her uneasiness, by its pricking her, when she turned her body suddenly in a particular direction. It was difficult, at first, for me to feel the substance she mentioned, but at length I got it between my thumb and finger. It appeared about the length and size of a common darning-needle. I advised her to have it taken out, and she readily consented to it, but as it was not convenient to have it done that day, the next day was agreed upon for the purpose. In the night, however, of the same day, I was called to her again, and found her suffering under decided symptoms of tetanus. She was thrown convulsively back, and her jaws were locked. I had now no doubt, at all, that the foreign substance she had mentioned, and which I had distinctly felt in the morning, was the exciting cause of her present symptoms. I therefore concluded not to defer the operation until the next morning, as we had previously agreed to do, but to remove the substance, whatever it might be, immediately, although obviously much more inconvenient doing it in the night, than in the day.

Accordingly I cut down and took out a piece about an inch and a half long, but sensible that I had not got the whole; and upon farther examination, I discovered and took out another piece, about an eighth of an inch long. These substances proved to be pieces of small *brass wire.*

The spasms continued through the night. They came on very regularly about once an hour, and continued for fifteen or twenty minutes, when they went off entirely, and she could speak, and move herself in every direction, as usual.

During the paroxysms of clonic spasms, the two varieties of Tetanus, denominated Opisthotonos and Trismus, were exhibited in as perfect a manner as I have ever witnesed them in any case that has come under my observation.

Feb. 3d.—I asked Dr. Warren to visit Mrs. A. in consultation.—The paroxysms still continued in full force, and as she was confident, from the pricking sensation she felt, that a portion of the wire still remained in her side, Dr. W. made a large incision for the purpose of ascertaining the fact, and of removing any fragment that might remain. The attempt proved ineffectual, as no vestige of the wire could be found. During the operation, a bubble of air was seen to come up from the lungs. This circumstance is mentioned here, as it may be of some importance should we attempt to solve the problem that naturally presents itself, viz., how came this wire here? Brass wire is an article not in common use among females—needles *are*; and the appearance of a needle in almost any part of the body would not be a circumstance, of very uncommon occurrence, it being well known that needles sometimes penetrate the skin without producing sufficient sensation to attract notice; and pass to extreme parts of the body with little or no inconvenience to the subject of their perambulations.

Feb. 4th.—Paroxysms still continued at irregular intervals. She was easily affected by opium. One grain would keep off the spasms for several hours. Musk also had a favorable effect, and was used as an auxiliary to the opium.

5th.—Paroxysms continued as on the 4th, at irregular intervals.

6th.—Mrs. A. had had no return of spasms since 3 o'clock, P. M., of the preceding day, about which time she felt something fall, as she thinks, from the external wound (made by cutting for the wire) down into the stomach. Prescribed ol. ricini.

7th.—There had been no recurrence of the spasms since the 5th, at 3 o'clock, P. M. The oil given the preceding day had operated very well, but owing to negligence, the dejections were not examined with sufficient care to determine satisfactorily, whether, or not, any unusual substance had passed.

8th.—She raised blood this morning.

9th.—She raised blood again.

17th.—Mrs. A. went on very well from last date. She told me, however, that the preceding night she had been kept awake by a pain in her right arm. Formerly, when quite young, she run a needle into her right thumb, which was broken off. The part was cut out at the time, but ever since, Mrs. A. told me that she had at times experienced pain extending from the right thumb to the right shoulder, very similar to what she suffered the night before.

19th.—Complained of a pricking sensation in the bowels, which she attributed to the wire passing down through the intestines; for she was fully confident that the sensation she felt on the 5th inst. was occasioned by a portion of the wire falling from the external wound down into her stomach.

21st.—Mrs. A. had had a slight spasm the preceding night. The right arm and shoulder were drawn back for a short time, but the paroxysm soon went off, and she was as well on this day as usual.

June 22d, 1831.—It is now nearly six months since Mrs. A.'s attack, and she has had but one slight symptom of tetanus since the 5th of February last. She has recently returned from a journey to the south, and from what I can learn, her health is better than it has been for several years.

I ought to mention that six weeks previous to my being called to her, as she was turning in bed, she felt something break in her side. She supposed it was the *bone*, for at that time she thought the moveable substance in her side, a splinter of bone from the ribs, as mentioned above. She was very faint for some time after this took place, and had not recovered her usual degree of health on the 31st of January last, the day on which she was attacked with this formidable complaint.

As a subject of curiosity, it would be some satisfaction to know how brass wire came to be in this woman's side without her knowledge.

"The thing is neither strange nor rare,
But how the d—l came it *there!*"

She distinctly recollects, that about a year before she perceived it, in attempting to swallow a piece of bread, she found great difficulty, and afterwards spit up blood from the throat. Her family also distinctly remember the circumstance, and the baker was dismissed immediately afterwards—not merely in consequence of this circumstance, but because she had frequently found dirt and other foreign substances in his bread before.

Is it not possible that a suspender wire, or a fragment of a tattered wire sieve, should by accident have gotten into the bread, and thus been swallowed? By the contractions of the stomach, or some other unknown cause, might not a portion of the wire have been straightened and propelled through the coats of this organ, the lower part of the lung, and the intercostal muscles, and, by the obstruction of the skin, been turned into a horizontal position, whilst the other extremity of it remained in the stomach? Could not the sensation of something breaking, which she felt some weeks before her recent illness, have been caused by a separation of the part thus protruded, from that in the stomach? and could not the sensation, which she afterwards experienced, on the 5th of February (when the spasms left her), of something falling down into the stomach from the external incision, have been the disengagement of this evil, which, having nothing to support it (the part protruded having been taken out), fell into the stomach, and afterwards caused, in its passage through the intestines, that pricking sensation which she complained of? This is all hypothesis, and I regret that I have not been able to ascertain in a more satisfactory manner the modus operandi of this important piece of wire.—From all the facts and circumstances of the case, however, I am induced to believe that it was, in the first instance, taken into the stomach.

*Boston, June* 23, 1831.

32. *On a New Practice in Acute and Chronic Rheumatism.* By J. K. Mitchell, M. D., *one of the attending Physicians of the Pennnsylvania Hospital.*—In the autumn of 1827, a patient affected with caries of the spine, was suddenly attacked with all the usual symptoms of acute rheumatism of the lower extremities. One ankle, and the knee of the opposite leg tumefied, red, hot, and painful, afforded as fair a specimen of that disease in its acute stage as is usually met with.—The usual treatment by leeches, purgatives, and cooling diaphoretics, with evaporating lotions, had the effect of transferring the symptoms to the other ankle and knee, and finally to the hip. Disappointed in the treatment, I began to suspect that the cause of the irritation might lie in the affected spine. The difficulty of cure, the transfer of irritation from one part of the lower extremities to another, without any sensible diminution of disease, and the fact of the existence of caries in the lumbar vertebræ, which lie near the origin of the nerves of the lower extremities, rendered probable the opinion, that in the spinal marrow lay the cause of this apparently indomitable and migratory inflammation. Under this impression, I caused leeches to be applied to the lumbar curve, and followed them by a blister, placed on the same spot. Relief promptly followed these remedies, and the pain ceasing to be felt in the limbs, was perceived only in the immediate vicinity of the spinal curve. After the blistered surface recovered its cuticle, a few leeches placed over the diseased spine removed the pain, and left the patient in the usual state of indifferent health attendant on such forms of spinal disease.

Striking as were the benefits of the applications made to the spine in a case of apparent inflammatory rheumatism, they did not lead my mind at the time, to the general conclusions which, viewing the case as I now do, they ought to have suggested.

In the beginning of the ensuing winter, another case of a similar kind peesented itself. A little female patient, having curvature of the cervical vertebræ, was attacked in the night with severe pain in the wrist, attended with redness, tumefaction, and heat. As on the appearance of these symptoms, the pain in the neck, to which she was accustomed, subsided, I easily persuaded myself of the spinal origin of this inflammation, and accordingly applied leeches to the cervical spine, with the effect of procuring a prompt solution of the disease of the wrist.

This case led me very naturally to the reflection that, perhaps other cases of rheumatism might have an origination in the *medulla spinalis*, and depend on an irritation of that important organ. In the following spring an opportunity of testing by practice the truth of this opinion presented itself. William Curran, a respectable livery stable keeper in Marshall's Court, had been for upwards of two years afflicted with a rheumatism of the lower extremities, which gradually deprived him of the use of his limbs, and finally confined him to his chamber. Regular medical aid, and many empirical remedies had been procured, without an abatement of the pain, which became at length almost intolerable.

On my first visit I found him in his room, in a paroxysm of pain.—His legs were swollen from knee to ankle, and the enlargement of the periosteum and integuments, gave to the anterior face of the tibia an unnatural prominence. In that place the pain and tenderness on pressure, were particularly developed. He was also suffering severely from pain in the scalp, which had existed for a short time previously, and was at length almost insupportable. Along with these symptoms appeared the usual febrile action with its concomitants.

Notwithstanding the significant hints given by the spine-cases referred to, I treated this case for a time in the usual manner—depleted freely, purged actively, blistered the head, and having caused an abatement of fever, administered corrosive sublimate and decoction of sarsaparilla. Defeated in all my efforts, I at length suggested to my patient the possibility that his disease was so unmanageable because we had not applied our remedies to the true seat of disease, and that by addressing our measures to the spine, success might yet be found.—Accordingly on the 16th of February, 1828, nine days after my first visit, I had him *cupped at the back of the neck*, and as he could not bear any more direct depletion, inserted a large seton over the *lumbar spinal region.* The cupping, followed by blisters to the back of the neck, relieved his head, and as soon as the seton began to suppurate freely, his legs became more comfortable. From the 25th, nine days after the insertion of the seton, I visited him but seldom, although I had seen him once or twice a day until that period. Indeed, I paid him but seven visits after the 25th. The last was on the 30th of March.—Soon afterwards he resumed his usual pursuits, and about the beginning of June the seton was removed. Since that time he has not had a return of his complaint, and is at the date of this paper, in the full and virgorous exercise of all his physical faculties.

I could scarcely doubt as to the cause of the cure in this case, because the treatment applied to the spine was that alone which had not already been fully and fairly tried, either by me or those who had preceded me. Indeed, the last applications were made with some hope of success, and the grounds of that hope were expressed to the patient, who was fully persuaded that the spinal treatment was the chief, if not the sole agent of restoration.

No other well-marked case of rheumatism presented itself in my private circle of practice, until in the winter of 1830, Mr. Teale's work on neuralgic diseases reached this country and began to attract towards the spinal marrow a greater share of medical attention. Although in his essay, I found nothing *directly* calculated to sustain me in the opinion I felt disposed to adopt concerning the spinal origin of rheumatism, I rose from its perusal with increased confidence in that opinion, and resolved experimentally to examine its truth. The first *well-marked* case of simple inflammatory rheumatism which subsequently presented itself, was the following:

Robert Gordon, well known as the carrier of Poulson's Daily Advertiser, fifty-six years of age, of vigorous constitution and active habits, was the subject of the attack. Observing a severe pain in his right heel and ankle, immediately followed by redness, heat and tumefaction, he caused himself to be largely bled, and took some salts and magnesia. On the following day the pain and swelling increased, and the ankle and knee of the opposite limb becoming similarly affected, he was confined to bed.

On the 3d day my first visit was made. The patient had then a full, strong, frequent pulse, flushed face, dry skin, whitened tongue, and complained much of the severity of the pain in his legs, and of his incapacity of enduring the slightest pressure or motion. As he had already been purged and had used a lotion, I directed the application of seventeen cups to the lumbar region, so as to abstract twelve or sixteen ounces of blood.

Next morning found the pain almost entirely gone, does not complain of moderate pressure, and is able to move his legs without inconvenience. Ordered a draught of salts and magnesia, with an evaporating lotion of camphor in alcohol.

3d day. Pain in legs scarcely perceptible, but the shoulders, elbows, and wrists, are beginning to exhibit marks of severe inflammation, expressed by pain, tumefaction, heat, and redness. Ordered twelve cups to the cervical spine.

4th day. The patient sits up, complains of stiffness but no pain except in one wrist, and that very slight. Directed Epsom salt and magnesia.

5th day. Finding nothing for which to prescribe, arranged the patient's diet, recommended the occasional use of aperients, and took leave of the case.

Called on the 10th to inquire into results, and found that there had been no return of disease.

Since that time a very severe winter has passed, during which the subject of this report has continued in his customary health, and in the pursuit of his usual employments.

The reader will, in the above case perceive, that the general bleeding, though very copious, proved of no service, and that the large local depletion of the lumbar region, benefitted solely that part of the disease which lay at the peripheral extremities of the nerves, supplied by the lower end of the spinal marrow. The inflammation in the upper extremities continued afterwards in progress, and was arrested only when cups were placed over the cervical end of the spinal column.

The whole case exhibits a fine exemplification of the difference in the character and extent of the influence of general and topical depletion, and proves that *local blood-letting is most potent when applied to that part of the spine, which supplies with nerves the parts in a state of active inflammation.*

As I feel, in common with the profession, a greater confidence in Hospital reports, especially when made by those who are not by interest or reputation blinded or misled, I shall present the history of some cases treated after the new method, as drawn up at my request, by Dr. Stewardson and Dr. Norris, the resident physicians of the Pennsylvania Hospital.

(Here follow eight cases of Rheumatism treated in a similar manner, with speedy relief in most of them, and benefit in all.)

Although other cases might be cited in confirmation of the views here taken, I have not leisure at this time to digest and arrange them. At no very distant period I hope to be able to bring the subject more fully before the profession. I may observe in general, that, as far as I now recollect, only two cases of apparent rheumatism, have in my hands, either in private practice, or in the Pennsylvania Hospital, resisted the treatment recommended in this paper, and both of *them* were in reality *neuralgia*, and exhibited no traces of inflammation.—One of them was an affection severely painful, located in the bottom of the heel, the other was gastric and intercostal.

The preference given to local depletion over other local measures, arose from the greater apparent success and promptness of its action, which scarcely left any thing to be desired: but cases will occur in which other measures must be used, and in which, perhaps, all measures will fail. We are warranted, however, in declaring our conviction, that few failures will happen in thus treating *acute* rheumatism, and that success will diminish, as passing through *chronic* rheumatism, we enter on the ground of *neuralgia*, a disease which sometimes spontaneously disappears; but is scarcely ever, in this city, cured by merely *medical* means. The art of the surgeon occasionally subdues it, and the physician often allays, but seldom removes it. Being paroxysmal, and often slumbering for weeks or months, it is not unfrequently mastered in appearance, though seldom cured in reality.—*Amer. Jour. Med. Sciences, May*, 1831.

33. *Colchicum in Rheumatism.*—The following letter from Mr. Tweedie, of Guy's Hospital, London, to the Medical Gazette of that city, gives the result of some trials of powdered colchicum root, in doses of 4 grs. every 4 hours—6 grs. every 6 hours, &c. &c., each dose combined with 20 or 30 grs. of Epsom salts, and a little magnesia. As the practice has been long common in this country, it is pleasant to find its success confirmed by an English physician of so much note as Dr. Addison.

Though colchicum, says Mr. Tweedie, has long maintained a very respectable rank in the list of our remedial agents for the cure of rheumatism, yet from time to time there have been some who have doubted its efficacy; and even when used with the happiest effects, the result has not been so speedy as to satisfy the sanguine expectations of many of its supporters. Much of this discrepency of opinion is doubtless ascribable to the notorious fact, that the fluid preparations of the drug are compounded in many different ways by various practitioners—a circumstance of itself sufficient to produce great uncertainty of effect, and in many cases complete disappointment.

Another great cause of failure and uncertainty may perhaps consist in this, that the remedial principle of the drug is probably not entirely taken up by the menstrua in either of our pharmacopœial preparations; so that when the vinum, or the acetum colchici, disappoints our expectations, we are scarcely justified in condemning the drug as useless, whose specific principle has, under such circumstances, never been administered at all, or very partially. To obviate these objections, it becomes necessary to administer the remedy *in substance*; and it is to show the benefit of this plan of exhibition that I have ventured to trouble you with these remarks.

The form of application most commonly, I believe, adopted, and which has for some time past been practiced by Dr. Bright in this hospital, is the vinum colchici, combined with so much magnesia and Epsom salt as might suffice to procure several stools daily. Now, this, though hitherto the most successful practice, was yet found to fail so frequently—to seem, in fact, so often inert, that Dr. Addison, our then clinical physician, was happy to avail himself of the evidence of Dr. Jackson, of Boston, in the United States, who was attending our clinical wards as a pupil, and who stated that in the hospital at Boston it was customary to administer the colchicum (for the cure of rheumatism) in substance, to the extent of ℨss. in twenty-four hours, combined with a little magnesia and salts, with such uniform good effect, that he had never had the opportunity of seeing any other plan of treatment called for or adopted, nor was depletion ever premised. Dr. Addison accordingly determined to give the remedy a fair trial, and the following cases, briefly related, will show with what effect.

[Four cases are here related, in all which it was successful, and the letter concludes with the following remarks.]

The sum and substance of what has been already said respecting the treatment of rheumatism, has, I believe, pretty well established the fact that colchicum is its best remedy; and I think the above cases (only a portion of those similarly treated by Dr. Addison in our clinical ward) afford proofs of the powers of the drug, such as are seldom witnessed from the exhibition of any of its artificial preparations. When administered as above described, in doses proportioned to the age and strength of the patient, it for the most part exercises an influence on the system at once marked, decided, and beneficial. After four or five doses have been taken, this influence begins to show itself: the pulse, which was before hard and frequent, diminishes in number very remarkably; it becomes at the same time softer and more expanded, and there is a hesitation in its beat, truly characteristic. The pulse, in fact, resembles much that of oppression of the brain in its slowness and hesitation, but none of its hardness. With this there is a sense of vertigo and dizziness, especially when the patient attempts to sit up. The pupils are sometimes dilated; there is occasionally, but not always, nausea; and the patient complains of uneasy griping sensations in the abdomen, which are chiefly referred to the hypogastric region, and always aggravated during the act of either of the evacuations. The stools are for the most part characteristic; they are of a peculiar loose, yellow nature, such as are seldom, I believe, seen under other circumstances.

In proportion as these symptoms indicate the influence of the colchicum on the system, the rheumatic pain becomes diminished, the swelling and inflammation subside, and in a very brief space of time indeed (as evidenced by the above-related cases,) the disease is at an end. The remedy being now discontinued, all its symptoms gradually decline, leaving the patient quite convalescent, and with a good appetite. But this remedy is by no means infallible.

When administered so as to make the impression on the system above detailed, and when the number of stools procured by its combination with the magnesia and salts does not exceed three or four daily, a rapid improvement takes place; but there are individuals who cannot be placed under this favorable influence. The exceptions to which I allude consist of those cases where the mucous membrane of the bowels is so irritable, that sufficient doses of colchicum cannot be administered without inducing excessive purging; and it has been found in our clinical ward, that when such purging takes place, the system manifests none of the symptoms above mentioned, as indicative of the influence of the medicine. In such cases, it would appear that it expended its whole powers on the bowels, and became removed from the body by the action of purging, before it had time to affect the geeral nervous and vascular systems in the peculiar manner on which seems to depend its favorable operation. These exceptions, however, constitute only a minority; and I know of no rule by which they may *a priori* be known. Dr. Addison gave the colchicum in nearly every case of acute rheumatism indiscriminately; if purging supervened, he found no difficulty in arresting it by means of Dover's powder, &c., and in continuing the treatment accordingly by other remedies.

